# Supplementary material for: Tempo and mode of morphological evolution are decoupled from latitude in birds
Source: PLoS Biol. 2021 Aug 24;19(8):e3001270. doi: 10.1371/journal.pbio.3001270 (PMC8384433; doi:10.1371/journal.pbio.3001270)
Supplement: S1 Text — (DOCX) [file pbio.3001270.s001.docx]

**S1 Appendix: Supplementary Methods**

*Parameter estimation in two-regime models*

To verify that the model fitting tools we developed for two-regime

models accurately estimate parameters, we conducted a simulation study for each of the models. In this study, we simulated one hundred trees with 50 tips under a Yule process using the pbtree function in the R package phytools [1]. We then simulated two regimes along the tree, one containing 30 lineages and the other containing 20 lineages, using the make.simmap function in phytools. We then simulated traits from the root to the tips of the phylogenies across a range of parameter values (S1 Table), setting the trait value at the root to 0. Finally, we fit the model for the generating process to each simulated dataset and compared the simulated and estimated parameter values.

In each case, the maximum likelihood estimates were reasonable approximations of the simulated parameter values (S2 Fig.). Importantly, the difference between the parameter values of the two regimes was particularly well estimated (S2 Fig. d,h,l,p). Preliminary analyses demonstrated that the ML estimates from the two-regime MC model were sensitive to the starting values of the optimization process. As a result, for each trait fit in our simulation and empirical analyses, we conducted fits using 5 different starting values and used the results from the fit with the highest likelihood.

*Inferences under two-regime BM, OU, and EB models*

To verify that data generated under two-regime models excluding competition (i.e., BM, OU, and EB models) are not best-fit by models that incorporate competition, we conducted a simulation study for each of the models. We used the same EB simulations as above, and using the same trees and regimes generated above, we simulated BM and OU traits along the phylogenies across a range of parameter values (S1 Table) in mvMORPH [2]. Finally, we fit the model for the generating process to each simulated dataset and conducted model selection as described in the main text (S8 Data).

Perhaps as a result of the relatively small number of lineages in each region, there are parts of parameter space where two-regime models are not better supported than single-regime BM models (S3 Fig). However, with the exception of the performance of single-regime MC models on two-regime EB simulations, the two-regime models simulated here generally do not lead to erroneously inferring the action of species interactions.

*Phylogenetic principal component analyses*

We constructed two sets of phylogenetic principal component axes [3]: one on the species mean of individually log-transformed values for bill length, height, and width data (bill pPCs), and another on the species mean of individually log-transformed values for wing, tarsus, and tail length (locomotion pPCs) (S2 Table, S3 Table). Each of these axes were constructed on the maximum clade credibility (MCC) bird phylogeny, trimmed to exclude species without data. We acknowledge that using pPCs in comparative analysis of traits involves a level of circularity, since BM evolution is an assumption of these transformations. Nevertheless, using phylogenetic, rather than non-phylogenetic, principal component analyses and conducting analyses across all resulting axes, rather than the first one or two, should help to ameliorate the statistical issues that are known to arise in analyses of trait evolution using PC scores [4].

*Accounting for within-species variation*

Ignoring within-species sources of variation in comparative studies can lead to bias in both parameter estimates and model selection [5–9]. This is, for instance, the case with observational error or uncertainty arising from the estimation of species mean phenotype from a finite sample of individuals and populations. On empirical datasets, observational error variance can be confounded by additional within-species component of variance resulting from instrumental errors, the use of a biased sample for estimating the mean as well as from short-lived causes [e.g., phenotypic plasticity, rapid genetic response to the environment or to fluctuating selection] that are not necessarily captured nor related to the (long-term) process used to model the evolution of the traits on the phylogeny. Holding constant this component of variance across species and assuming that it is independent between species, we can model interspecific trait values by decomposing it as a sum of a phylogenetic, unknown (and independent) intra-specific, and known observational error components:

$$\tilde{\boldsymbol{X}}=z_{0}+\delta+\gamma+\varepsilon$$

Where $\delta\sim\mathcal{N}\left( 0,\sigma_{phylo}^{2}\boldsymbol{D} \right), \gamma\sim\mathcal{N}\left( 0,\sigma_{intra}^{2}\boldsymbol{I} \right),$and $\varepsilon\sim\mathcal{N}\left( 0,\sigma_{error}^{2}\boldsymbol{I} \right)$, are all normally distributed random deviates with scale variance $\sigma_{phylo}^{2}$, $\sigma_{intra}^{2}$ and $\sigma_{error}^{2}$, ***I*** is an identity matrix, and ***D*** is the phylogenetic variance-covariance matrix implied by the evolutionary process used to model **X** in the main text. This decomposition corresponds to a linear mixed model [5] where the variance components $\sigma_{phylo}^{2}$ and $\sigma_{intra}^{2}$ are unknown and have to be estimated during the model fit while $\sigma_{error}^{2}$ is the known sampling variance estimated from multiple individual measurements per species (i.e., the squared standard errors). Given that the evolutionary processes considered here are all Gaussian and it is assumed that the intraspecific components of variance are normally distributed, estimation of all the parameters is done by maximizing the multivariate normal likelihood as generally done in comparative studies [2,10,11].

In our model fits, we input $\sigma_{error}^{2}$ for each of the traits. To calculate these values (i.e., the squared standard errors) for pPC scores, we projected individual, log-transformed measurements [12] into pPC space [13], of which there were on average 4-5 per species (see *Materials and methods*), and then calculated the species-level standard error directly on these values. For body mass, we obtained mean values from EltonTraits [14], using source data [15] where possible to calculate the standard error. When source data were not available, we calculated a standard deviation from the range of mass values when standard deviations were not reported [16] or using the averaged standard deviation across species when a sample size and mean were the only values available or for cases where measurements were only made on a single individual [6]. We then transformed standard error estimates to the log scale [17].

*Examining the potential impact of assuming continental-scale sympatry: further details*

To generate downsampled biogeographies, we first trimmed the clade level phylogeny into two separate trees (one for tropical lineages and one for temperate lineages). On each tree, we generated a bank of 100 stochastic maps for each clade simulating the evolution of a categorical trait meant to represent separate geographic regions within continents, using make.simmap in phytools [1] and an Mk model using rTraitDisc in ape [18]. This categorical trait had either two states in each of the tropical and temperate region (for scenario 1) or two states in the temperate region and three in the tropics (for scenario 2). We then joined the simulated traits across lineages to create a trait with 4 states (for scenario 1) or 5 states (for scenario 2). Finally, we merged the matrices indicating continental-scale sympatry (i.e., the ones used in our empirical analyses) with these simulated matrices such that any two lineages that are not in the same simulated state were set to ‘allopatric’ (i.e., $\mathbf{A}_{j,l}$ = 0) in the original biogeography matrix. In scenario 1, this meant that 50% of lineages that were treated as sympatric in our empirical analyses because they occurred on the same continent were set to be allopatric. In scenario 2, this meant that 50% of temperate lineages and 66.67% of tropical lineages that were treated as sympatric in our empirical analyses because they occurred on the same continent were set to be allopatric.

We then simulated traits under the parameter values in S8 Table across the banks of stochastic maps of downsampled biogeographies using the function simulateTipData in RPANDA [19].

**References**

1. Revell LJ. phytools: An R package for phylogenetic comparative biology (and other things). Methods Ecol Evol. 2012;3: 217–223.

2. Clavel J, Escarguel G, Merceron G. mvMORPH: an R package for fitting multivariate evolutionary models to morphometric data. Methods Ecol Evol.; 2015;6: 1311–1319.

3. Revell LJ. Size-correction and principal components for interspecific comparative studies. Evolution. Wiley Online Library; 2009;63: 3258–3268.

4. Uyeda JC, Caetano DS, Pennell MW. Comparative analysis of principal components can be misleading. Syst Biol. 2015;64: 677–689. doi:10.1093/sysbio/syv019

5. Housworth EA, Martins EP, Lynch M. The phylogenetic mixed model. Am Nat. The University of Chicago Press; 2004;163: 84–96.

6. Ives AR, Midford PE, Garland T. Within-species variation and measurement error in phylogenetic comparative methods. Syst Biol. 2007;56: 252–270. doi:10.1080/10635150701313830

7. Felsenstein J. Comparative methods with sampling error and within-species variation: contrasts revisited and revised. Am Nat. The University of Chicago Press; 2008;171: 713–725.

8. Silvestro D, Kostikova A, Litsios G, Pearman PB, Salamin N. Measurement errors should always be incorporated in phylogenetic comparative analysis. Methods Ecol Evol. 2015;6: 340–346. doi:10.1111/2041-210X.12337

9. Landis MJ, Schraiber JG. Pulsed evolution shaped modern vertebrate body sizes. Proc Natl Acad Sci.; 2017;114: 13224–13229.

10. Drury J, Clavel J, Manceau M, Morlon H. Estimating the effect of competition on trait evolution using maximum likelihood inference. Syst Biol. 2016;65: 700–710. doi:10.1017/CBO9781107415324.004

11. Manceau M, Lambert A, Morlon H. A unifying comparative phylogenetic framework including traits coevolving across interacting lineages. Syst Biol. 2017; https://doi.org/10.1093/sysbio/syw115.

12. Pigot AL, Sheard C, Miller ET, Bregman TP, Freeman BG, Roll U, et al. Macroevolutionary convergence connects morphological form to ecological function in birds. Nat Ecol Evol.; 2020; doi:10.1038/s41559-019-1070-4

13. Drury JP, Tobias JA, Burns KJ, Mason NA, Shultz AJ, Morlon H. Contrasting impacts of competition on ecological and social trait evolution in songbirds. PLoS Biol.; 2018;16: e2003563.

14. Wilman H, Belmaker J, Simpson J, de la Rosa C, Rivadeneira MM, Jetz W. EltonTraits 1.0: Species-level foraging attributes of the world’s birds and mammals. Ecology.; 2014;95: 2027.

15. Dunning Jr JB. CRC Handbook of Avian Body Masses. CRC Press; 2008.

16. Hozo SP, Djulbegovic B, Hozo I. Estimating the mean and variance from the median, range, and the size of a sample. BMC Med Res Methodol.; 2005;5: 13.

17. Clavel J, Morlon H. Accelerated body size evolution during cold climatic periods in the Cenozoic. Proc Natl Acad Sci. 2017;114: 4183–4188. doi:10.1073/pnas.1606868114

18. Paradis E. Analysis of Phylogenetics and Evolution with R. New York, NY: Springer; 2011.

19. Morlon H, Lewitus E, Condamine FL, Manceau M, Clavel J, Drury J. RPANDA: an R package for macroevolutionary analyses on phylogenetic trees. Methods Ecol Evol.; 2016;7: 589–597. doi:10.1111/2041-210X.12526

20. Freckleton RP, Harvey PH, Pagel M. Phylogenetic analysis and comparative data: a test and review of evidence. Am Nat.; 2002;160: 712–726.

21. Hadfield J. MCMCglmm course notes. See http//cran.r-project.org/web/packages/MCMCglmm/vignettes/CourseNotes.pdf. 2012;

22. Gelman A, Rubin DB, others. Inference from iterative simulation using multiple sequences. Stat Sci. Institute of Mathematical Statistics; 1992;7: 457–472.

23. Plummer M, Best N, Cowles K, Vines K. CODA: Convergence Diagnosis and Output Analysis for MCMC. R News. 2006;6: 7–11. Available: https://journal.r-project.org/archive/
