# Supplementary material for: Tempo and mode of morphological evolution are decoupled from latitude in birds
Source: PLoS Biol. 2021 Aug 24;19(8):e3001270. doi: 10.1371/journal.pbio.3001270 (PMC8384433; doi:10.1371/journal.pbio.3001270)
Supplement: S1 Table — (DOCX) [file pbio.3001270.s002.docx]

**S1 Table.** Parameters used for simulations generating datasets used to test two-regime models.

| **model** | ***σ*^2^ (tips)** | **regime 1 parameter** | **regime 2 parameter** |
| --- | --- | --- | --- |
|  |  |  |  |
| *TD-exponential (EB)* | 0.01 | -2.5 | -1 |
|  |  | -2.5 | -2.5 |
|  |  | -2.5 | -5 |
|  |  | -1 | -2.5 |
|  |  | -5 | -2.5 |
|  |  |  |  |
| *DD-linear* | 0.2 | -0.005 | 0.0025 |
|  |  | -0.005 | 0 |
|  |  | -0.005 | -0.0025 |
|  |  | -0.005 | -0.005 |
|  |  | -0.005 | -0.001 |
|  |  | 0.005 | -0.005 |
|  |  | 0 | -0.005 |
|  |  | -0.0025 | -0.005 |
|  |  | -0.005 | -0.005 |
|  |  | -0.001 | -0.005 |
|  |  |  |  |
| *DD-exponential* | 0.01 | -0.05 | 0.05 |
|  |  | -0.05 | 0 |
|  |  | -0.05 | -0.025 |
|  |  | -0.05 | -0.05 |
|  |  | -0.05 | -0.1 |
|  |  | 0.05 | -0.05 |
|  |  | 0 | -0.05 |
|  |  | -0.025 | -0.05 |
|  |  | -0.05 | -0.05 |
|  |  | -0.1 | -0.05 |
|  |  |  |  |
| *MC* | 0.05 | -0.5 | 0 |
|  |  | -0.5 | -0.25 |
|  |  | -0.5 | -0.5 |
|  |  | -0.5 | -1 |
|  |  | 0 | -0.5 |
|  |  | -0.25 | -0.5 |
|  |  | -0.5 | -0.5 |
|  |  | -1 | -0.5 |
|  |  |  |  |
| *BM* |  | 1 | 0.5 |
|  |  | 2 | 0.5 |
|  |  | 0.5 | 0.5 |
|  |  | 0.5 | 1 |
|  |  | 0.5 | 2 |
|  |  |  |  |
| *OU* | 0.5 (*α* = 2) | 0 | 1 |
|  | 0.5 (*α* = 2) | 0 | 2 |
|  | 0.5 (*α* = 0.5) | 0 | 1 |
|  | 0.5 (*α* = 0.5) | 0 | 2 |
|  |  |  |  |
