# Supplementary material for: Tempo and mode of morphological evolution are decoupled from latitude in birds
Source: PLoS Biol. 2021 Aug 24;19(8):e3001270. doi: 10.1371/journal.pbio.3001270 (PMC8384433; doi:10.1371/journal.pbio.3001270)
Supplement: S2 Table — (DOCX) [file pbio.3001270.s003.docx]

**S2 Table.** Description of morphological variables.

| **trait** | **description** |
| --- | --- |
| Body mass | ln-transformed body mass |
| Bill pPC1 | High scores = small overall beak size (highest score is *Tachornis phoenicobia*; lowest is *Buceros rhinoceros*) |
| Bill pPC2 | High scores = shape of beak is relatively long and thin (highest score is *Ensifera ensifera*; lowest is *Glaucidium mooreorum*) |
| Bill pPC3 | High scores = shape of beak is relatively narrow (highest score is *Chlorostilbon olivaresi*; lowest is *Caprimulgus maculosus*) |
| Locomotion pPC1 | High scores = small overall size (highest score is *Chaetocercus berlepschi*; lowest is *Leptoptilos dubius*) |
| Locomotion pPC2 | High scores = relatively long tail (highest score is *Eupetomena macroura*; lowest is *Grus americana*) |
| Locomotion pPC3 | High scores = relatively long wings and short tarsi (highest is *Panyptila sanctihieronymi*; lowest is *Stipiturus malachurus*) |
