# Supplementary material for: Tempo and mode of morphological evolution are decoupled from latitude in birds
Source: PLoS Biol. 2021 Aug 24;19(8):e3001270. doi: 10.1371/journal.pbio.3001270 (PMC8384433; doi:10.1371/journal.pbio.3001270)
Supplement: S3 Table — pPC, phylogenetic principal component. (DOCX) [file pbio.3001270.s004.docx]

**S3 Table.** Loadings for pPC axes of bill and locomotion measurements.

| **trait group** | **trait** | **pPC1** | **pPC2** | **pPC3** |
| --- | --- | --- | --- | --- |
| bill | ln(culmen length) | -0.86 | 0.51 | -0.07 |
|  | ln(bill width) | -0.89 | -0.33 | -0.3 |
|  | ln(bill depth) | -0.92 | -0.17 | 0.34 |
| locomotion | ln(wing length) | -0.83 | -0.28 | 0.48 |
|  | ln(tarsus length) | -0.78 | -0.56 | -0.28 |
|  | ln(tail length) | -0.92 | 0.38 | -0.07 |
