# Supplementary material for: Tempo and mode of morphological evolution are decoupled from latitude in birds
Source: PLoS Biol. 2021 Aug 24;19(8):e3001270. doi: 10.1371/journal.pbio.3001270 (PMC8384433; doi:10.1371/journal.pbio.3001270)
Supplement: S4 Table — Statistical support was measured as the mean Akaike weights of single-regime models (i.e., calculated from pool of single-regime models only), and relative support for a model with competition, defined as the maximum Akaike weight for a model with competition divided by the sum of this value and the maximum Akaike weight for a model without competition [max(MCwi, DDlin_wi, DDexp_wi)/((max(BMwi,OUwi,EBwi)+max(MCwi, DDlin_wi, DDexp_wi))], limiting analyses to clades with ≥ 50 tips (n = 66). Values indicated in bold are those that are significant after controlling for multiple testing (α = 0.05/7). λ indicates the MLE of the phylogenetic signal. BM, Brownian motion; DD, diversity-dependent; DDexp, exponential diversity-dependent; DDlin, linear diversity-dependent; EB, early burst; MC, matching competition; MLE, maximum likelihood estimate; OU, Ornstein–Uhlenbeck; PGLS, phylogenetic generalized least squares. (DOCX) [file pbio.3001270.s005.docx]

**S4 Table.** Phylogenetic generalised least-squares (PGLS) models of statistical support as a function of the latitudinal distribution (measured as the proportion of lineages with individuals that breed in tropical regions). Statistical support was measured as the mean Akaike weights of single-regime models (i.e., calculated from pool of single regime models only), and relative support for a model with competition, (defined as the maximum Akaike weight for a model with competition divided by the sum of this value and the maximum Akaike weight for a model without competition [max(MC_wi_, DD_lin­_wi_, DD_exp_wi_)/((max(BM_wi_,OU_wi_,EB_wi_)+max(MC_wi_, DD_lin­_wi_, DD_exp_wi_))], limiting analyses to clades with $\geq$ 50 tips (n =66). Values indicated in bold are those that are significant after controlling for multiple testing (α = 0.05/7). λ indicates the maximum likelihood estimate of the phylogenetic signal.

| **term** | **trait** | **estimate** | **std. error** | **t-value** | **p-value** | **λ** |
| --- | --- | --- | --- | --- | --- | --- |
| Akaike weight: BM | ln(mass) | -0.16 | 0.07 | -2.15 | 0.04 | 0 |
|  | bill pPC1 | -0.15 | 0.07 | -2.12 | 0.04 | 0 |
|  | bill pPC2 | -0.02 | 0.08 | -0.33 | 0.74 | 0 |
|  | bill pPC3 | 0.02 | 0.07 | 0.30 | 0.77 | 0 |
|  | locomotion pPC1 | -0.10 | 0.07 | -1.41 | 0.16 | 0 |
|  | locomotion pPC2 | 0.05 | 0.07 | 0.66 | 0.51 | 0 |
|  | locomotion pPC3 | -0.11 | 0.08 | -1.36 | 0.18 | 0 |
|  |  |  |  |  |  |  |
| Akaike weight: OU | ln(mass) | 0.01 | 0.05 | 0.21 | 0.83 | 0 |
|  | bill pPC1 | 0.02 | 0.07 | 0.34 | 0.74 | 0 |
|  | bill pPC2 | 0.05 | 0.08 | 0.61 | 0.54 | 0 |
|  | bill pPC3 | -0.05 | 0.07 | -0.71 | 0.48 | 0 |
|  | locomotion pPC1 | 0.02 | 0.04 | 0.44 | 0.66 | 0 |
|  | locomotion pPC2 | 0.04 | 0.08 | 0.48 | 0.63 | 0 |
|  | locomotion pPC3 | -0.1 | 0.05 | -2.09 | 0.04 | 0 |
|  |  |  |  |  |  |  |
| Akaike weight: EB | ln(mass) | 0.07 | 0.1 | 0.7 | 0.49 | 0 |
|  | bill pPC1 | -0.01 | 0.09 | -0.1 | 0.92 | 0 |
|  | bill pPC2 | -0.01 | 0.09 | -0.09 | 0.93 | 0.68 |
|  | bill pPC3 | -0.07 | 0.06 | -1.13 | 0.26 | 0 |
|  | locomotion pPC1 | -0.01 | 0.08 | -0.13 | 0.9 | 0 |
|  | locomotion pPC2 | -0.09 | 0.11 | -0.84 | 0.41 | 0.42 |
|  | locomotion pPC3 | -0.11 | 0.13 | -0.86 | 0.39 | 0.8 |
|  |  |  |  |  |  |  |
| Akaike weight: DDexp | ln(mass) | -0.09 | 0.1 | -0.97 | 0.33 | 0 |
|  | bill pPC1 | 0.09 | 0.09 | 1.07 | 0.29 | 1 |
|  | bill pPC2 | -0.01 | 0.13 | -0.11 | 0.91 | 0 |
|  | bill pPC3 | -0.13 | 0.1 | -1.32 | 0.19 | 0 |
|  | locomotion pPC1 | -0.04 | 0.11 | -0.36 | 0.72 | 0 |
|  | locomotion pPC2 | -0.1 | 0.12 | -0.9 | 0.37 | 0 |
|  | locomotion pPC3 | -0.19 | 0.11 | -1.78 | 0.08 | 0 |
|  |  |  |  |  |  |  |
| Akaike weight: DDlin | ln(mass) | -0.02 | 0.04 | -0.56 | 0.58 | 0 |
|  | bill pPC1 | -0.01 | 0.04 | -0.27 | 0.79 | 0 |
|  | bill pPC2 | 0.04 | 0.05 | 0.77 | 0.45 | 0 |
|  | bill pPC3 | 0.04 | 0.04 | 1.02 | 0.31 | 0 |
|  | locomotion pPC1 | -0.04 | 0.04 | -0.96 | 0.34 | 0.84 |
|  | locomotion pPC2 | 0.05 | 0.04 | 1.15 | 0.25 | 0 |
|  | locomotion pPC3 | 0.05 | 0.04 | 1.33 | 0.19 | 0 |
|  |  |  |  |  |  |  |
| Akaike weight: MC | ln(mass) | 0.2 | 0.15 | 1.36 | 0.18 | 0 |
|  | bill pPC1 | 0.12 | 0.15 | 0.81 | 0.42 | 0 |
|  | bill pPC2 | -0.02 | 0.14 | -0.12 | 0.9 | 0 |
|  | bill pPC3 | 0.2 | 0.12 | 1.64 | 0.11 | 0 |
|  | locomotion pPC1 | 0.16 | 0.15 | 1.05 | 0.3 | 0 |
|  | locomotion pPC2 | 0.05 | 0.13 | 0.35 | 0.73 | 0 |
|  | **locomotion pPC3** | **0.41** | **0.13** | **3.29** | **0.002** | **0** |
|  |  |  |  |  |  |  |
| relative support for a | ln(mass) | 0.08 | 0.14 | 0.53 | 0.6 | 0 |
| model with competition | bill pPC1 | 0.17 | 0.13 | 1.3 | 0.2 | 0 |
|  | bill pPC2 | 0 | 0.15 | 0 | 1 | 0 |
|  | bill pPC3 | 0.09 | 0.13 | 0.7 | 0.48 | 0 |
|  | locomotion pPC1 | 0.09 | 0.13 | 0.7 | 0.49 | 0 |
|  | locomotion pPC2 | -0.05 | 0.15 | -0.31 | 0.76 | 0 |
|  | locomotion pPC3 | 0.24 | 0.14 | 1.74 | 0.09 | 0 |
