# Supplementary material for: Tempo and mode of morphological evolution are decoupled from latitude in birds
Source: PLoS Biol. 2021 Aug 24;19(8):e3001270. doi: 10.1371/journal.pbio.3001270 (PMC8384433; doi:10.1371/journal.pbio.3001270)
Supplement: S5 Table — The index of relative support for any 2-regime model was calculated using max(2-regime Akaike weight)/(max(2-regime Akaike weight)+max(single-regime Akaike weight)); other model specific indices were calculated using max(2-regime Akaike weight for specified model)/(max(2-regime Akaike weight for specified model) + max(single-regime Akaike weight for specified model)). For each model, this index was transformed by subtracting 0.5 such that negative estimates indicate support for a single-regime model and positive values equal support for a 2-regime model. Values indicated in bold are those that are significant after controlling for multiple testing (α = 0.05/7). For all significant cases, the single-regime version of the model was supported over the 2-regime version. λ indicates the MLE of the phylogenetic signal. MLE, maximum likelihood estimate; PGLS, phylogenetic generalized least squares. (DOCX) [file pbio.3001270.s006.docx]

**S5 Table.** Intercept-only PGLS models fit to indices of support for two regimes models for each trait (for cases where N $\geq$ 50; *n* = 59). The index of relative support for any two-regime model was calculated using max(two regime Akaike weight)/(max(two regime Akaike weight)+max(single regime Akaike weight)); other, model specific indices were calculated using max(two regime Akaike weight for specified model)/ (max(two regime Akaike weight for specified model) + max(single regime Akaike weight for specified model)). For each model, this index was transformed by subtracting 0.5 such that negative estimates indicate support for a single-regime model and positive values equal support for a two-regime model. Values indicated in bold are those that are significant after controlling for multiple testing (α = 0.05/7). For all significant cases, the single-regime version of the model was supported over the two-regime version. λ indicates the maximum likelihood estimate of the phylogenetic signal.

| **response variable** | **trait** | **estimate** | **s.e.** | ***t*-value** | ***p*-value** | ***λ*** |
| --- | --- | --- | --- | --- | --- | --- |
| relative support for | ln(mass) | 0.04 | 0.04 | 0.89 | 0.38 | 0.11 |
| any two-regime model | bill pPC1 | 0.03 | 0.03 | 1.10 | 0.28 | 0 |
|  | bill pPC2 | -0.01 | 0.02 | -0.47 | 0.64 | 0 |
|  | bill pPC3 | 0.00 | 0.02 | 0.04 | 0.97 | 0 |
|  | locomotion pPC1 | 0.01 | 0.02 | 0.40 | 0.69 | 0 |
|  | locomotion pPC2 | 0.07 | 0.03 | 2.53 | 0.01 | 0 |
|  | locomotion pPC3 | 0.04 | 0.03 | 1.34 | 0.18 | 0 |
|  |  |  |  |  |  |  |
| relative support for | ln(mass) | 0.00 | 0.02 | 0.17 | 0.86 | 0 |
| two-regime BM model | bill pPC1 | 0.00 | 0.03 | 0.18 | 0.86 | 0 |
|  | bill pPC2 | -0.02 | 0.02 | -0.75 | 0.46 | 0 |
|  | **bill pPC3** | **-0.06** | **0.02** | **-3.84** | **0.0003** | **0** |
|  | locomotion pPC1 | -0.02 | 0.02 | -0.89 | 0.38 | 0 |
|  | locomotion pPC2 | -0.02 | 0.02 | -1.01 | 0.31 | 0 |
|  | locomotion pPC3 | 0.01 | 0.02 | 0.39 | 0.70 | 0 |
|  |  |  |  |  |  |  |
| relative support for | **ln(mass)** | **-0.10** | **0.02** | **-5.82** | **< 0.0001** | **0** |
| two-regime OU model | bill pPC1 | -0.06 | 0.02 | -2.75 | 0.01 | 0 |
|  | **bill pPC2** | **-0.09** | **0.02** | **-4.81** | **< 0.0001** | **0** |
|  | **bill pPC3** | **-0.07** | **0.02** | **-3.66** | **0.0006** | **0** |
|  | **locomotion pPC1** | **-0.08** | **0.02** | **-3.95** | **0.0002** | **0** |
|  | locomotion pPC2 | -0.02 | 0.03 | -0.83 | 0.41 | 0 |
|  | locomotion pPC3 | 0.06 | 0.03 | 2.1 | 0.04 | 0 |
|  |  |  |  |  |  |  |
| relative support for | ln(mass) | 0.04 | 0.06 | 0.61 | 0.54 | 0.38 |
| two-regime EB model | bill pPC1 | 0.02 | 0.03 | 0.77 | 0.44 | 0 |
|  | bill pPC2 | 0.03 | 0.03 | 0.95 | 0.34 | 0 |
|  | bill pPC3 | -0.02 | 0.02 | -0.65 | 0.52 | 0 |
|  | locomotion pPC1 | 0.00 | 0.02 | 0.00 | 1.00 | 0 |
|  | locomotion pPC2 | 0.03 | 0.03 | 1.17 | 0.25 | 0 |
|  | locomotion pPC3 | 0.01 | 0.03 | 0.23 | 0.82 | 0 |
|  |  |  |  |  |  |  |
| relative support for | ln(mass) | -0.04 | 0.08 | -0.43 | 0.67 | 0.72 |
| two-regime DD_exp_ model | bill pPC1 | 0.00 | 0.02 | 0.19 | 0.85 | 0 |
|  | bill pPC2 | 0.01 | 0.06 | 0.23 | 0.82 | 0.42 |
|  | bill pPC3 | -0.03 | 0.02 | -1.26 | 0.21 | 0 |
|  | locomotion pPC1 | -0.01 | 0.03 | -0.29 | 0.78 | 0 |
|  | locomotion pPC2 | 0.03 | 0.03 | 1.24 | 0.22 | 0 |
|  | locomotion pPC3 | -0.03 | 0.02 | -1.04 | 0.30 | 0 |
|  |  |  |  |  |  |  |
| relative support for | ln(mass) | -0.08 | 0.03 | -2.73 | 0.01 | 0 |
| two-regime DD_lin_ model | bill pPC1 | -0.01 | 0.04 | -0.25 | 0.80 | 0 |
|  | bill pPC2 | 0.09 | 0.11 | 0.76 | 0.45 | 0.55 |
|  | bill pPC3 | -0.06 | 0.04 | -1.54 | 0.13 | 0 |
|  | locomotion pPC1 | -0.03 | 0.03 | -1.01 | 0.32 | 0 |
|  | locomotion pPC2 | -0.09 | 0.03 | -2.68 | 0.01 | 0 |
|  | **locomotion pPC3** | **-0.12** | **0.04** | **-3.48** | **0.001** | **0** |
|  |  |  |  |  |  |  |
| relative support for | ln(mass) | -0.08 | 0.07 | -1.20 | 0.24 | 0.65 |
| two-regime MC model | bill pPC1 | -0.08 | 0.07 | -1.28 | 0.21 | 0.52 |
|  | **bill pPC2** | **-0.16** | **0.02** | **-7.67** | **< 0.0001** | **0** |
|  | **bill pPC3** | **-0.19** | **0.02** | **-10.98** | **< 0.0001** | **0** |
|  | locomotion pPC1 | -0.12 | 0.05 | -2.30 | 0.03 | 0.41 |
|  | **locomotion pPC2** | **-0.16** | **0.02** | **-7.96** | **< 0.0001** | **0** |
|  | **locomotion pPC3** | **-0.18** | **0.02** | **-8.02** | **< 0.0001** | **0** |
|  |  |  |  |  |  |  |
