# Supplementary material for: Tempo and mode of morphological evolution are decoupled from latitude in birds
Source: PLoS Biol. 2021 Aug 24;19(8):e3001270. doi: 10.1371/journal.pbio.3001270 (PMC8384433; doi:10.1371/journal.pbio.3001270)
Supplement: S6 Table — All comparisons were conducted on clades with ≥ 50 species (n = 66). Note: One outlier was removed from the exponential diversity dependence analysis of bill pPC2. Values indicated in bold are those that are significant after controlling for multiple testing (α = 0.05/7). λ indicates the MLE of the phylogenetic signal. MLE, maximum likelihood estimate; PGLS, phylogenetic generalized least squares; pPC, phylogenetic principal component. (DOCX) [file pbio.3001270.s007.docx]

**S6 Table.** PGLS models comparing the observed latitudinal distribution (measured as the proportion of lineages with individuals that breed in tropical regions) of clade-by-trait level fits with the mean maximum likelihood estimates (across fits conducted on a bank of stochastic maps of ancestral biogeography) of the strength of species interactions in single-regime models incorporating competition. All comparisons were conducted on clades with $\geq$ 50 species (*n* = 66). Note: one outlier was removed from the exponential diversity dependence analysis of bill pPC2. Values indicated in bold are those that are significant after controlling for multiple testing (α = 0.05/7). λ indicates the maximum likelihood estimate of the phylogenetic signal.

| **model (parameter)** | **trait** | **estimate** | **std. error** | **t-value** | **p-value** | **λ** |
| --- | --- | --- | --- | --- | --- | --- |
| DDexp (slope) | ln(mass) | -0.01 | 0.05 | -0.28 | 0.78 | 1 |
|  | bill pPC1 | 0 | 0.03 | 0.04 | 0.97 | 0.42 |
|  | bill pPC2 | -1.67 | 1.19 | -1.41 | 0.16 | 0.35 |
|  | bill pPC3 | -0.87 | 1.27 | -0.68 | 0.5 | 0 |
|  | locomotion pPC1 | -0.18 | 0.5 | -0.36 | 0.72 | 1 |
|  | locomotion pPC2 | -1.97 | 1.16 | -1.7 | 0.09 | 0 |
|  | locomotion pPC3 | 0.38 | 0.26 | 1.48 | 0.14 | 0 |
|  |  |  |  |  |  |  |
| DDlin (slope) | ln(mass) | -9.92E-05 | 9.64E-05 | -1.03 | 0.31 | 0.56 |
|  | bill pPC1 | -5.18E-05 | 5.58E-05 | -0.93 | 0.36 | 0.53 |
|  | bill pPC2 | -3.51E-06 | 1.11E-05 | -0.32 | 0.75 | 0 |
|  | bill pPC3 | -4.26E-07 | 4.07E-06 | -0.1 | 0.92 | 0 |
|  | locomotion pPC1 | -2.82E-05 | 3.77E-05 | -0.75 | 0.46 | 0 |
|  | locomotion pPC2 | 8.97E-06 | 9.19E-06 | 0.98 | 0.33 | 0.47 |
|  | locomotion pPC3 | 2.86E-06 | 3.38E-06 | 0.85 | 0.4 | 0 |
|  |  |  |  |  |  |  |
| MC (S) | ln(mass) | -0.06 | 0.03 | -2.15 | 0.04 | 0 |
|  | bill pPC1 | -0.06 | 0.04 | -1.52 | 0.13 | 0 |
|  | bill pPC2 | -0.18 | 0.1 | -1.9 | 0.06 | 0.58 |
|  | bill pPC3 | -0.12 | 0.06 | -2.01 | 0.05 | 0 |
|  | locomotion pPC1 | -0.06 | 0.03 | -1.87 | 0.07 | 0 |
|  | locomotion pPC2 | 0.01 | 0.04 | 0.26 | 0.8 | 0 |
|  | **locomotion pPC3** | **-0.1** | **0.02** | **-4.1** | **0.0001** | **0** |
|  |  |  |  |  |  |  |
