# Supplementary material for: Tempo and mode of morphological evolution are decoupled from latitude in birds
Source: PLoS Biol. 2021 Aug 24;19(8):e3001270. doi: 10.1371/journal.pbio.3001270 (PMC8384433; doi:10.1371/journal.pbio.3001270)
Supplement: S8 Table — For each evolutionary model (a: MC, b: DDexp, c: DDlin), the mean (across fits conducted on a bank of stochastic maps of ancestral biogeography and stochastic maps of breeding range) of the log-transformed ratio of the absolute value of parameter estimates for tropical taxa to that of temperate taxa (ln(|par_tropical|/|par_temperate|)) was the response variable in the intercept-only PGLS model. Negative estimates, therefore, indicate that the impact of competition is estimated to be higher in temperate regions, whereas positive estimates indicate that competition is higher in the tropics. Values indicated in bold are those that are significant after controlling for multiple testing (α = 0.05/7). λ indicates the MLE of the phylogenetic signal. DDexp, exponential diversity-dependent; DDlin, linear diversity-dependent; ML, maximum likelihood; MLE, maximum likelihood estimate; PGLS, phylogenetic generalized least squares. (DOCX) [file pbio.3001270.s009.docx]

**S8 Table.** Intercept-only PGLS models linear regressions fit to tropical/temperate comparisons of maximum likelihood parameter estimates of the strength of species interactions in two-regime models (for cases where N$\geq$100) (*n* = 34) for each trait. For each evolutionary model (a: MC, b: DD_exp_, c: DD_lin_), the mean (across fits conducted on a bank of stochastic maps of ancestral biogeography and stochastic maps of breeding range) of the log-transformed ratio of the absolute value of parameter estimates for tropical taxa to that of temperate taxa (ln(|par_tropical|/|par_temperate|)) was the response variable in the intercept-only PGLS model. Negative estimates, therefore, indicate that the impact of competition is estimated to be higher in temperate regions, whereas positive estimates indicate that competition is higher in the tropics. Values indicated in bold are those that are significant after controlling for multiple testing (α = 0.05/7). λ indicates the maximum likelihood estimate of the phylogenetic signal.

| **response variable** | **model term** | **estimate** | **s.e.** | ***t*-value** | ***p*-value** | **λ** |
| --- | --- | --- | --- | --- | --- | --- |
| MC | ln(mass) | -1.43 | 1.16 | -1.23 | 0.23 | 0 |
| (ln(\|S_tropical_\|/ \|S_temperate_\|)) | bill pPC1 | -0.62 | 1.23 | -0.5 | 0.62 | 0 |
|  | bill pPC2 | -1.9 | 0.89 | -2.13 | 0.04 | 0 |
|  | bill pPC3 | -1.15 | 1.06 | -1.08 | 0.29 | 0 |
|  | locomotion pPC1 | -0.78 | 1.21 | -0.64 | 0.52 | 0 |
|  | locomotion pPC2 | 2.43 | 1.16 | 2.1 | 0.04 | 0 |
|  | locomotion pPC3 | -1.06 | 1.29 | -0.82 | 0.42 | 0 |
|  |  |  |  |  |  |  |
| DD_exp_ | ln(mass) | -0.08 | 0.3 | -0.28 | 0.78 | 0 |
| (ln(\|r_tropical_\|/ \|r_temperate_\|)) | bill pPC1 | 0.23 | 0.34 | 0.66 | 0.51 | 0 |
|  | bill pPC2 | 0.22 | 0.36 | 0.6 | 0.55 | 0 |
|  | bill pPC3 | -0.4 | 0.35 | -1.13 | 0.27 | 0 |
|  | locomotion pPC1 | -0.22 | 0.33 | -0.68 | 0.5 | 0 |
|  | locomotion pPC2 | -0.46 | 0.26 | -1.78 | 0.08 | 0 |
|  | locomotion pPC3 | 0.21 | 0.35 | 0.6 | 0.55 | 0 |
|  |  |  |  |  |  |  |
| DD_lin_ | ln(mass) | -0.39 | 0.58 | -0.67 | 0.51 | 0 |
| (ln(\|b_tropical_\|/ \|b_temperate_\|)) | bill pPC1 | -0.05 | 0.56 | -0.08 | 0.93 | 0 |
|  | bill pPC2 | -1.12 | 0.96 | -1.16 | 0.25 | 0 |
|  | bill pPC3 | -0.85 | 1.1 | -0.77 | 0.44 | 0 |
|  | locomotion pPC1 | -0.11 | 0.66 | -0.17 | 0.87 | 0 |
|  | locomotion pPC2 | -0.81 | 0.91 | -0.89 | 0.38 | 0 |
|  | locomotion pPC3 | -1.47 | 1.06 | -1.39 | 0.17 | 0 |
|  |  |  |  |  |  |  |
