# Supplementary material for: Tempo and mode of morphological evolution are decoupled from latitude in birds
Source: PLoS Biol. 2021 Aug 24;19(8):e3001270. doi: 10.1371/journal.pbio.3001270 (PMC8384433; doi:10.1371/journal.pbio.3001270)
Supplement: S9 Table — (DOCX) [file pbio.3001270.s010.docx]

**S9 Table.** Zero-intercept mixed-effect linear model with a random effect for clade identity fit to the proportion of lineages pairs in each clade that are sympatric in each continent.

| **response variable** | **model term** | **estimate** | **s.e.** | ***t*-value** |
| --- | --- | --- | --- | --- |
| Proportion of species pairs sympatric | W Palearctic | 0.74 | 0.04 | 19.82 |
|  | E Palearctic | 0.54 | 0.03 | 16.43 |
|  | W Nearctic | 0.70 | 0.04 | 19.74 |
|  | E Nearctic | 0.66 | 0.04 | 18.59 |
|  | Madagascar | 0.68 | 0.04 | 16.65 |
|  | Africa | 0.36 | 0.03 | 11.44 |
|  | South America | 0.34 | 0.03 | 10.66 |
|  | Central America | 0.41 | 0.03 | 13.2 |
|  | India | 0.63 | 0.03 | 19.59 |
|  | SE Asia | 0.40 | 0.03 | 12.89 |
|  | Oceania | 0.37 | 0.04 | 10.45 |
