# Supplementary material for: Tempo and mode of morphological evolution are decoupled from latitude in birds
Source: PLoS Biol. 2021 Aug 24;19(8):e3001270. doi: 10.1371/journal.pbio.3001270 (PMC8384433; doi:10.1371/journal.pbio.3001270)
Supplement: S10 Table — (DOCX) [file pbio.3001270.s011.docx]

**S10 Table.** Intercept-only mixed-effect linear model with a random effect for clade identity fit to the proportion of lineages pairs that are sympatric in each clade.

| **response variable** | **model term** | **estimate** | **s.e.** | ***t*-value** |
| --- | --- | --- | --- | --- |
| Proportion of species pairs sympatric | intercept | 0.50 | 0.02 | 26.32 |
|  |  |  |  |  |
