# Supplementary material for: Tempo and mode of morphological evolution are decoupled from latitude in birds
Source: PLoS Biol. 2021 Aug 24;19(8):e3001270. doi: 10.1371/journal.pbio.3001270 (PMC8384433; doi:10.1371/journal.pbio.3001270)
Supplement: S11 Table — (DOCX) [file pbio.3001270.s012.docx]

**S11 Table.** Linear model fit to the proportion of lineages pairs that are sympatric as a function of the absolute value of midpoint latitude for species pairs.

| **response variable** | **model term** | **estimate** | **s.e.** | ***z*-value** | ***p*-value** |
| --- | --- | --- | --- | --- | --- |
| Proportion of species pairs sympatric | intercept | -0.98 | 0.01 | -126.46 | < 0.001 |
|  | abs(midpoint latitude) | 0.02 | 0.0004 | 43.12 | < 0.001 |
