# Supplementary material for: Tempo and mode of morphological evolution are decoupled from latitude in birds
Source: PLoS Biol. 2021 Aug 24;19(8):e3001270. doi: 10.1371/journal.pbio.3001270 (PMC8384433; doi:10.1371/journal.pbio.3001270)
Supplement: S12 Table — Values were chosen based on MLEs from single-regime MC models. MC, matching competition; MLE, maximum likelihood estimate. (DOCX) [file pbio.3001270.s013.docx]

**S12 Table.** Simulation parameters used in simulation study to explore the statistical power of two-regime MC models under realistic levels of sympatry. Values were chosen based on maximum likelihood estimates (MLEs) from single-regime MC models.

| **clade** | **simulation parameters** | | | |
| --- | --- | --- | --- | --- |
|  | *σ^2^* | *S*_tropical_ | *S*_temperate_ | S_tropical_:S_temperate_ |
| Cracidae.0  (MLE for single-regime MC:  *σ^2^* = 0.00464021,  *S* = -0.1269343) | 0.00464021 | -0.1269343 | -0.1269343 | 1:1 |
|  | 0.00464021 | -0.3808029 | -0.1269343 | 3:1 |
|  | 0.00464021 | -0.1904015 | -0.1269343 | 3:2 |
|  | 0.00464021 | -0.1269343 | -0.1904015 | 2:3 |
|  | 0.00464021 | -0.1269343 | -0.3808029 | 1:3 |
| Nectariniidae.0  (MLE for single-regime MC:  *σ^2^* = 0.00000676,  *S* = -0.1626235) | 0.00000676 | -0.1626235 | -0.1626235 | 1:1 |
|  | 0.00000676 | -0.4878705 | -0.1626235 | 3:1 |
|  | 0.00000676 | -0.2439352 | -0.1626235 | 3:2 |
|  | 0.00000676 | -0.1626235 | -0.2439352 | 2:3 |
|  | 0.00000676 | -0.1626235 | -0.4878705 | 1:3 |
| Picidae.1  (MLE for single-regime MC:  *σ^2^* = 0.00684987,  *S* = -0.0691007) | 0.00684987 | -0.0691007 | -0.0691007 | 1:1 |
|  | 0.00684987 | -0.207302 | -0.0691007 | 3:1 |
|  | 0.00684987 | -0.103651 | -0.0691007 | 3:2 |
|  | 0.00684987 | -0.0691007 | -0.103651 | 2:3 |
|  | 0.00684987 | -0.0691007 | -0.207302 | 1:3 |
