# Supplementary material for: Tempo and mode of morphological evolution are decoupled from latitude in birds
Source: PLoS Biol. 2021 Aug 24;19(8):e3001270. doi: 10.1371/journal.pbio.3001270 (PMC8384433; doi:10.1371/journal.pbio.3001270)
Supplement: S13 Table — For DD models, parameter estimates are the mean estimates across fits conducted on a bank of stochastic maps of ancestral biogeography. λ indicates the MLE of the phylogenetic signal. DD, diversity-dependent; MLE, maximum likelihood estimate; PGLS, phylogenetic generalized least squares. (DOCX) [file pbio.3001270.s014.docx]

**S13 Table.** PGLS analyses of maximum likelihood estimates of evolutionary rates in single-regime model fits (*n* = 135) as a function of the latitudinal distribution (measured as the proportion of lineages with individuals that breed in tropical regions). For diversity-dependent models, parameter estimates are the mean estimates across fits conducted on a bank of stochastic maps of ancestral biogeography. λ indicates the maximum likelihood estimate of the phylogenetic signal.

| **model (parameter)** | **trait** | **estimate** | **std. error** | ***t*-value** | ***p*-value** | **λ** |
| --- | --- | --- | --- | --- | --- | --- |
| BM (σ^2^) | ln(mass) | -0.0015 | 0.0024 | -0.64 | 0.52 | 0 |
|  | bill pPC1 | -0.0017 | 0.0014 | -1.18 | 0.24 | 0 |
|  | bill pPC2 | -0.00016 | 0.00028 | -0.56 | 0.58 | 0 |
|  | bill pPC3 | -8.58E-05 | 8.93E-05 | -0.96 | 0.34 | 0 |
|  | locomotion pPC1 | 0.0014 | 0.0013 | 1.07 | 0.29 | 0 |
|  | locomotion pPC2 | 0.00029 | 0.00035 | 0.81 | 0.42 | 0 |
|  | locomotion pPC3 | -5.71E-05 | 9.15E-05 | -0.62 | 0.53 | 0 |
|  |  |  |  |  |  |  |
| DDexp (maximum σ^2^ | ln(mass) | -0.0022 | 0.0025 | -0.86 | 0.39 | 0 |
| at the tips) | bill pPC1 | -0.0025 | 0.0015 | -1.71 | 0.09 | 0 |
|  | bill pPC2 | -0.00029 | 0.00026 | -1.11 | 0.27 | 0 |
|  | bill pPC3 | -0.00011 | 0.00013 | -0.84 | 0.4 | 0.86 |
|  | locomotion pPC1 | 0.0016 | 0.0018 | 0.92 | 0.36 | 0 |
|  | locomotion pPC2 | 0.00062 | 0.00052 | 1.19 | 0.24 | 0 |
|  | locomotion pPC3 | -0.00011 | 0.00012 | -0.94 | 0.35 | 0 |
|  |  |  |  |  |  |  |
| DDlin (maximum σ^2^ | ln(mass) | -0.0024 | 0.0025 | -0.98 | 0.33 | 0 |
| at the tips) | bill pPC1 | -0.0027 | 0.0015 | -1.79 | 0.08 | 0 |
|  | bill pPC2 | -0.00021 | 0.00027 | -0.78 | 0.44 | 0 |
|  | bill pPC3 | -0.00017 | 8.37E-05 | -1.97 | 0.05 | 0 |
|  | locomotion pPC1 | 0.00093 | 0.0016 | 0.6 | 0.55 | 0 |
|  | locomotion pPC2 | 0.00035 | 0.00046 | 0.76 | 0.45 | 0 |
|  | locomotion pPC3 | -0.00016 | 9.50E-05 | -1.71 | 0.09 | 0 |
|  |  |  |  |  |  |  |
| EB (σ^2^ at the tips) | ln(mass) | -0.0013 | 0.0021 | -0.63 | 0.53 | 0 |
|  | bill pPC1 | -0.00073 | 0.0010 | -0.72 | 0.47 | 0 |
|  | bill pPC2 | -0.00015 | 0.00019 | -0.82 | 0.42 | 0 |
|  | bill pPC3 | -5.20E-05 | 7.57E-05 | -0.69 | 0.49 | 0 |
|  | locomotion pPC1 | 0.0013 | 0.0012 | 1.11 | 0.27 | 0 |
|  | locomotion pPC2 | 0.00032 | 0.00035 | 0.92 | 0.36 | 0 |
|  | locomotion pPC3 | -5.11E-05 | 8.95E-05 | -0.57 | 0.57 | 0 |
