# Supplementary material for: Tempo and mode of morphological evolution are decoupled from latitude in birds
Source: PLoS Biol. 2021 Aug 24;19(8):e3001270. doi: 10.1371/journal.pbio.3001270 (PMC8384433; doi:10.1371/journal.pbio.3001270)
Supplement: S14 Table — For DD models, the rate parameter was calculated as the mean comparisons between parameter estimates across fits conducted on a bank of stochastic maps of ancestral biogeography and stochastic maps of breeding range. Note: One outlier was removed from the linear diversity dependence analysis of locomotion pPC2 as it was >2 orders of magnitude larger than the next largest value. Values indicated in bold are those that are significant after controlling for multiple testing (α = 0.05/7). λ indicates the MLE of the phylogenetic signal. ML, maximum likelihood; MLE, maximum likelihood estimate; PGLS, phylogenetic generalized least squares; pPC, phylogenetic principal component. (DOCX) [file pbio.3001270.s015.docx]

**S14 Table.** Intercept-only PGLS models fit to the difference between tropical and temperate maximum likelihood parameter estimates of evolutionary rates in two-regime models, fit separately for each trait (*n* = 71 for ln.mass and *n* = 70 for other traits). For DD models, the rate parameter was calculated as the mean comparisons between parameter estimates across fits conducted on a bank of stochastic maps of ancestral biogeography and stochastic maps of breeding range. Note: one outlier was removed from the linear diversity dependence analysis of locomotion pPC2 as it was > 2 orders of magnitude larger than the next largest value. Values indicated in bold are those that are significant after controlling for multiple testing (α = 0.05/7). λ indicates the maximum likelihood estimate of the phylogenetic signal.

| **response variable** | **model term** | **estimate** | **s.e.** | ***t*-value** | ***p*-value** | **λ** |
| --- | --- | --- | --- | --- | --- | --- |
|  |  |  |  |  |  |  |
| BM (σ^2^_tropical_ - σ^2^_temperate_) | ln(mass) | -0.0037 | 0.0029 | -1.26 | 0.21 | 0.48 |
|  | bill pPC1 | 1.5E-05 | 0.00052 | 0.03 | 0.98 | 0 |
|  | bill pPC2 | -0.00023 | 8.0E-05 | -2.88 | 0.01 | 0 |
|  | bill pPC3 | 2.40E-05 | 4.0E-05 | 0.60 | 0.55 | 0 |
|  | locomotion pPC1 | 0.00017 | 0.00042 | 0.40 | 0.69 | 0 |
|  | locomotion pPC2 | 0.0001 | 0.00016 | 0.67 | 0.51 | 0 |
|  | **locomotion pPC3** | **-0.00014** | **4.30E-05** | **-3.29** | **0.002** | **0** |
|  |  |  |  |  |  |  |
| EB (σ^2^_tropical_ - σ^2^_temperate_) | ln(mass) | -0.0014 | 0.00086 | -1.66 | 0.10 | 0 |
|  | bill pPC1 | 0.00013 | 0.00048 | 0.27 | 0.79 | 0 |
|  | bill pPC2 | -0.00015 | 5.6E-05 | -2.64 | 0.01 | 0 |
|  | bill pPC3 | 0.00011 | 0.0001 | 1.09 | 0.28 | 0.48 |
|  | locomotion pPC1 | 0.00022 | 0.00035 | 0.64 | 0.53 | 0 |
|  | locomotion pPC2 | 0.00012 | 0.00014 | 0.84 | 0.40 | 0 |
|  | **locomotion pPC3** | **-0.00011** | **3.4E-05** | **-3.23** | **0.002** | **0** |
|  |  |  |  |  |  |  |
| DD_exp_ (σ^2^_tropical_ - σ^2^_temperate_) | ln(mass) | -0.0023 | 0.0014 | -1.59 | 0.12 | 0 |
|  | bill pPC1 | -0.002 | 0.0093 | -0.22 | 0.83 | 1 |
|  | **bill pPC2** | **-0.00032** | **7.5E-05** | **-4.22** | **< 0.0001** | **0** |
|  | bill pPC3 | 0.00023 | 0.00026 | 0.88 | 0.38 | 0.64 |
|  | locomotion pPC1 | -0.00075 | 0.0015 | -0.52 | 0.61 | 0 |
|  | locomotion pPC2 | -0.00015 | 0.001 | -0.14 | 0.89 | 0.5 |
|  | locomotion pPC3 | -0.00043 | 0.00031 | -1.42 | 0.16 | 0.5 |
|  |  |  |  |  |  |  |
| DD_lin_ (σ^2^_tropical_ - σ^2^_temperate_) | ln(mass) | -0.0053 | 0.003 | -1.80 | 0.08 | 0.29 |
|  | bill pPC1 | -0.0018 | 0.0017 | -1.06 | 0.30 | 0.26 |
|  | **bill pPC2** | **-0.00023** | **6.4E-05** | **-3.59** | **0.0006** | **0** |
|  | bill pPC3 | -6.8E-06 | 4.3E-05 | -0.16 | 0.88 | 0 |
|  | locomotion pPC1 | -0.00044 | 0.00052 | -0.85 | 0.40 | 0 |
|  | locomotion pPC2 | 7.8E-05 | 0.00024 | 0.33 | 0.74 | 0 |
|  | locomotion pPC3 | -0.00011 | 8.4E-05 | -1.34 | 0.19 | 0 |
|  |  |  |  |  |  |  |
