# Supplementary material for: Tempo and mode of morphological evolution are decoupled from latitude in birds
Source: PLoS Biol. 2021 Aug 24;19(8):e3001270. doi: 10.1371/journal.pbio.3001270 (PMC8384433; doi:10.1371/journal.pbio.3001270)
Supplement: S15 Table — Values indicated in bold are those that are significant after controlling for multiple testing (α = 0.05/7). λ indicates the MLE of the phylogenetic signal. BM, Brownian motion; MLE, maximum likelihood estimate; PGLS, phylogenetic generalized least squares. (DOCX) [file pbio.3001270.s016.docx]

**S15 Table.** PGLS models comparing the observed latitudinal distribution (measured as the proportion of lineages with individuals that breed in tropical regions) of clade-by-trait level fits (*n* = 135) with the log-transformed error (calculated as the sum of the maximum likelihood estimated error parameter and the clade-level mean squared standard error) in single-regime Brownian motion models. Values indicated in bold are those that are significant after controlling for multiple testing (α = 0.05/7). λ indicates the maximum likelihood estimate of the phylogenetic signal.

| **trait** | **intercept** | **estimate** | **std. error** | ***t*-value** | ***p*-value** | **λ** |
| --- | --- | --- | --- | --- | --- | --- |
|  |  |  |  |  |  |  |
| **ln(mass)** | **-3.92** | **-1.41** | **0.5** | **-2.83** | **0.0054** | **0** |
| bill pPC1 | -4.49 | -0.38 | 0.46 | -0.82 | 0.41 | 0.37 |
| bill pPC2 | -5.55 | -0.29 | 0.33 | -0.86 | 0.39 | 0.46 |
| bill pPC3 | -6.38 | 0.24 | 0.28 | 0.83 | 0.41 | 0.55 |
| locomotion pPC1 | -4.88 | -0.53 | 0.43 | -1.22 | 0.23 | 0 |
| locomotion pPC2 | -5.45 | -0.49 | 0.35 | -1.39 | 0.17 | 0.4 |
| locomotion pPC3 | -6.99 | 0.04 | 0.31 | 0.13 | 0.9 | 0 |
