# Supplementary material for: Tempo and mode of morphological evolution are decoupled from latitude in birds
Source: PLoS Biol. 2021 Aug 24;19(8):e3001270. doi: 10.1371/journal.pbio.3001270 (PMC8384433; doi:10.1371/journal.pbio.3001270)
Supplement: S16 Table — Values indicated in bold are those that are significant after controlling for multiple testing (α = 0.05/7). λ indicates the MLE of the phylogenetic signal. BM, Brownian motion; MLE, maximum likelihood estimate; PGLS, phylogenetic generalized least squares. (DOCX) [file pbio.3001270.s017.docx]

**S16 Table.** PGLS models comparing the observed latitudinal distribution (measured as the proportion of lineages with individuals that breed in tropical regions) of clade-by-trait level fits (*n* = 135) with the maximum likelihood parameter estimates of evolutionary rates in single-regime Brownian motion models that do not account for observational error. Values indicated in bold are those that are significant after controlling for multiple testing (α = 0.05/7). λ indicates the maximum likelihood estimate of the phylogenetic signal.

| **model (parameter)** | **trait** | **estimate** | **std. error** | ***t*-value** | ***p*-value** | **λ** |
| --- | --- | --- | --- | --- | --- | --- |
|  |  |  |  |  |  |  |
| BM (σ^2^) | **ln(mass)** | **-0.012** | **0.0035** | **-3.38** | **0.0009** | **0.95** |
|  | bill pPC1 | -0.0064 | 0.0032 | -1.99 | 0.05 | 0 |
|  | bill pPC2 | -0.00087 | 0.00047 | -1.86 | 0.06 | 0.47 |
|  | bill pPC3 | -0.00024 | 0.00028 | -0.88 | 0.38 | 0.73 |
|  | locomotion pPC1 | -0.00065 | 0.0024 | -0.27 | 0.79 | 0 |
|  | locomotion pPC2 | -0.00022 | 0.00061 | -0.36 | 0.72 | 0 |
|  | **locomotion pPC3** | **-0.00075** | **0.00021** | **-3.54** | **0.0006** | **1** |
|  |  |  |  |  |  |  |
