# Supplementary material for: Tempo and mode of morphological evolution are decoupled from latitude in birds
Source: PLoS Biol. 2021 Aug 24;19(8):e3001270. doi: 10.1371/journal.pbio.3001270 (PMC8384433; doi:10.1371/journal.pbio.3001270)
Supplement: S17 Table — Values indicated in bold are those that are significant after controlling for multiple testing (α = 0.05/7). λ indicates the MLE of the phylogenetic signal. BM, Brownian motion; MLE, maximum likelihood estimate; PGLS, phylogenetic generalized least squares. (DOCX) [file pbio.3001270.s018.docx]

**S17 Table.** Intercept only PGLS models fit to the mean difference (across stochastic maps of tropical and temperate living) in MLE estimates of tropical and temperate rates (from two-rate BM models that do not account for observational error) (*n* = 71 for log-transformed body mass, 70 for other traits). Values indicated in bold are those that are significant after controlling for multiple testing (α = 0.05/7). λ indicates the maximum likelihood estimate of the phylogenetic signal.

| **response variable** | **model term** | **estimate** | **s.e.** | ***t*-value** | ***p*-value** | **λ** |
| --- | --- | --- | --- | --- | --- | --- |
| BM (σ^2^_tropical_ - σ^2^_temperate_) | **ln(mass)** | **-0.0039** | **0.0012** | **-3.15** | **0.002** | **0** |
|  | bill pPC1 | -0.0026 | 0.0016 | -1.61 | 0.11 | 0 |
|  | bill pPC2 | -0.00067 | 0.00024 | -2.76 | 0.01 | 0 |
|  | bill pPC3 | 4.1E-05 | 9.1E-05 | 0.45 | 0.65 | 0 |
|  | locomotion pPC1 | -0.0016 | 0.0013 | -1.21 | 0.23 | 0 |
|  | locomotion pPC2 | -0.00062 | 0.00032 | -1.96 | 0.05 | 0 |
|  | **locomotion pPC3** | **-0.00027** | **7.9E-05** | **-3.38** | **0.001** | **0** |
|  |  |  |  |  |  |  |
