# Supplementary material for: Tempo and mode of morphological evolution are decoupled from latitude in birds
Source: PLoS Biol. 2021 Aug 24;19(8):e3001270. doi: 10.1371/journal.pbio.3001270 (PMC8384433; doi:10.1371/journal.pbio.3001270)
Supplement: S18 Table — The factors predicting which clades support models with competition, as revealed by PGLMMs fit to single-regime clade-by-trait fits (n = 924) with a categorical variable indicating (a) that the MC was the modal best-fit model (i.e., the most common best-fit model across fits conducted on a bank of stochastic maps of ancestral biogeography) (n = 166) or (b) that the DDexp model was the model best-fit model (n = 66) (S12 Data). The influence of the phylogeny was estimated from the random effect component of the PGLMM—the phylogenetic intraclass correlation coefficient is analogous to the λ parameter (often referred to as “phylogenetic signal”) estimated from PGLS models [91]. To facilitate parameter exploration, we rescaled all predictor variables using z-transformations. We used an uninformative, inverse Wishart distribution as a prior for the random effects, a flat prior for the fixed effects, and fixed the residual variance at 1 [92]. To fit the models, we ran an MCMC chain for at least 5 × 10^5^ generations, recording model results every 100 generations and ignoring the first 5 × 10^3^ generations as burn-in. We fit each model 4 times and merged the 4 chains after verifying convergence both visually and using Gelman–Rubin diagnostics in the R-package coda [93,94]. Estimates and credibility intervals are therefore calculated from the pooled posterior distributions. The pMCMC (an MCMC derived p-value calculated as 2 times the proportion of estimates in either the positive or negative portion (whichever is smaller) of the posterior distribution) is presented from one chain. DD, diversity-dependent; DDexp, exponential diversity-dependent; MC, matching competition; MCMC, Markov chain Monte Carlo; PGLMM, phylogenetic generalized linear mixed model; PGLS, phylogenetic generalized least squares; pMCMC, Markov chain Monte Carlo. (DOCX) [file pbio.3001270.s019.docx]

**S18 Table.** The factors predicting which clades support models with competition, as revealed by Phylogenetic Generalised Linear Mixed Models (PGLMMs) fit to single-regime clade-by-trait fits (*n* = 924) with a categorical variable indicating (a) that the matching competition was the modal best fit model (i.e., the most common best fit model across fits conducted on a bank of stochastic maps of ancestral biogeography) (*n* = 166) or (b) that the exponential diversity dependent model was the model best fit model (*n* = 66) (S12 Data). The influence of the phylogeny was estimated from the random effect component of the PGLMM—the phylogenetic intraclass correlation coefficient is analogous to the λ parameter (often referred to as ‘phylogenetic signal’) estimated from phylogenetic generalized least squares models [20]. To facilitate parameter exploration, we rescaled all predictor variables using *z*-transformations. We used an uninformative, inverse Wishart distribution as a prior for the random effects, a flat prior for the fixed effects, and fixed the residual variance at 1 [21]. To fit the models, we ran an MCMC chain for at least 5 x 10^5^ generations, recording model results every 100 generations and ignoring the first 5 x 10^3^ generations as burn-in. We fit each model four times and merged the four chains after verifying convergence both visually and using Gelman-Rubin diagnostics in the R-package coda [22,23]. Estimates and credibility intervals are therefore calculated from the pooled posterior distributions. The pMCMC (an MCMC derived *p*-value calculated as two times the proportion of estimates in either the positive or negative portion (whichever is smaller) of the posterior distribution) is presented from one chain.

| **response variable** | **model term** | **median estimate** | **(95% CI)** | ***p*MCMC** |
| --- | --- | --- | --- | --- |
| (a) MC best fit == 1 | (intercept) | -1.68 | (-3.38, 0.09) | 0.06 |
| *λ* = 0.51 | clade age | -0.02 | (-0.45, 0.40) | 0.94 |
| (95% CI = 0.32, 0.67) | **clade richness** | **0.80** | **(0.42 , 1.23)** | **< 0.001** |
|  | **max prop. species coexisting** | **0.76** | **(0.31 , 1.26)** | **0.002** |
|  | prop. species strongly territorial | 0.21 | (-0.21, 0.64) | 0.32 |
|  | **prop. species single-strata habitats** | **-0.55** | **(-1.10, 0.07)** | **0.02** |
|  | diet diversity | 0.06 | (-0.35, 0.46) | 0.77 |
|  |  |  |  |  |
| (b) DD_exp_ best fit == 1 | **(intercept)** | **-3.41** | **(-4.11, -2.70)** | **< 0.001** |
| *λ* = 0.06 | clade age | -0.09 | (-0.44, 0.24) | 0.61 |
| (95% CI = 0.0002, 0.34) | **clade richness** | **0.51** | **(0.20, 0.84)** | **0.002** |
|  | **max prop. species coexisting** | **-0.71** | **(-1.11, -0.32)** | **< 0.001** |
|  | prop. species strongly territorial | 0.15 | (-0.21, 0.49) | 0.42 |
|  | prop. species single-strata habitats | -0.37 | (-0.83, 0.03) | 0.07 |
|  | diet diversity | -0.08 | (-0.42, 0.24) | 0.60 |
|  |  |  |  |  |
