# Supplementary figures and images for: Tempo and mode of morphological evolution are decoupled from latitude in birds
Source: PLoS Biol. 2021 Aug 24;19(8):e3001270. doi: 10.1371/journal.pbio.3001270 (PMC8384433; doi:10.1371/journal.pbio.3001270)

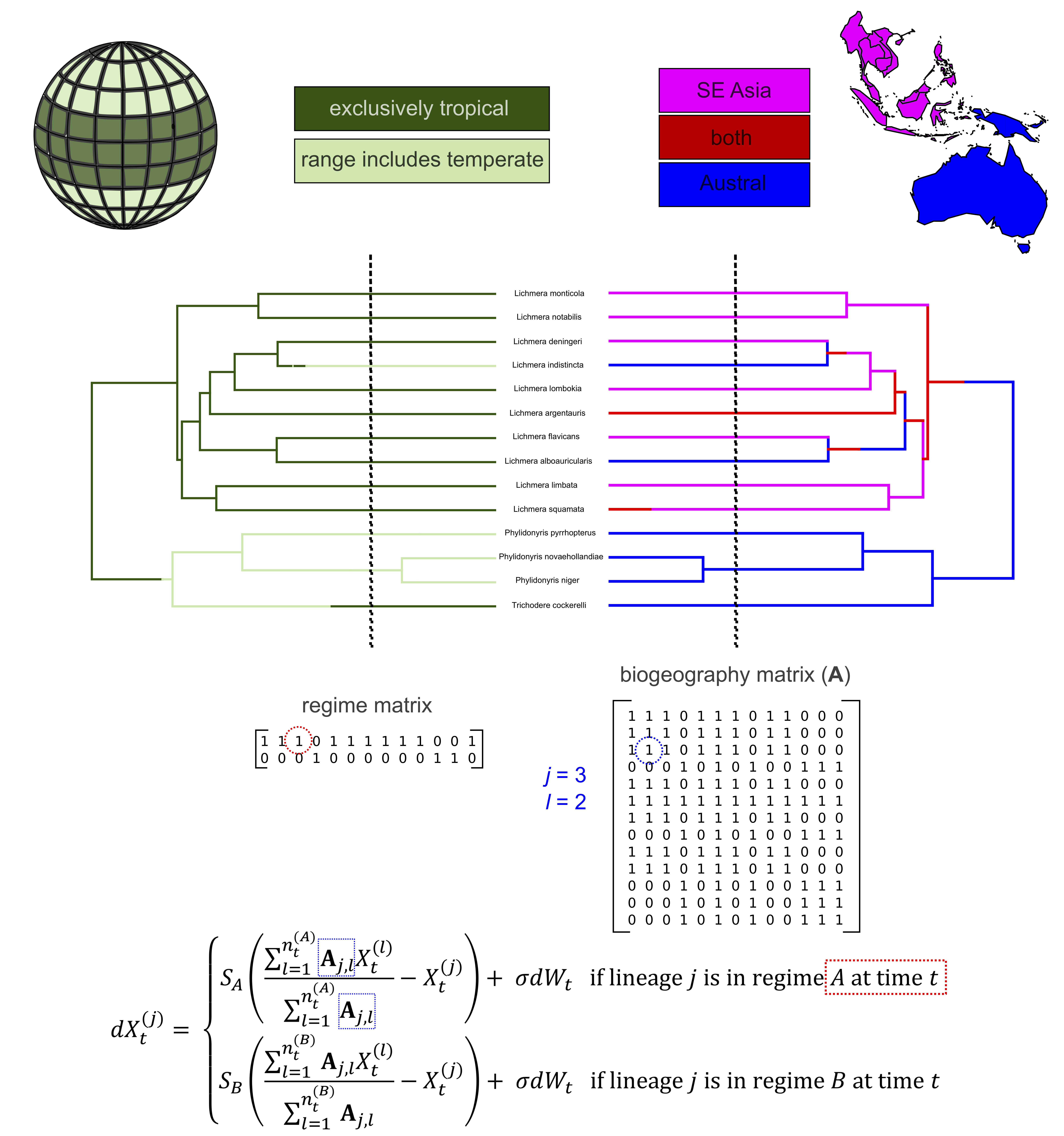

Supplement: S1 Fig — We combine a matrix of the presence or absence of each lineage in tropical/temperate regions (“regime matrix”) with a matrix of biogeography (denoted “A”) to identify the competitive regime of each lineage and the identity of other lineages with which the focal lineage is able to interact with. Blue and red colors in the lower panel denote correspondence between the formula and the biogeography matrix (A) and the regime matrix, respectively. (TIFF) [file pbio.3001270.s020.tiff]

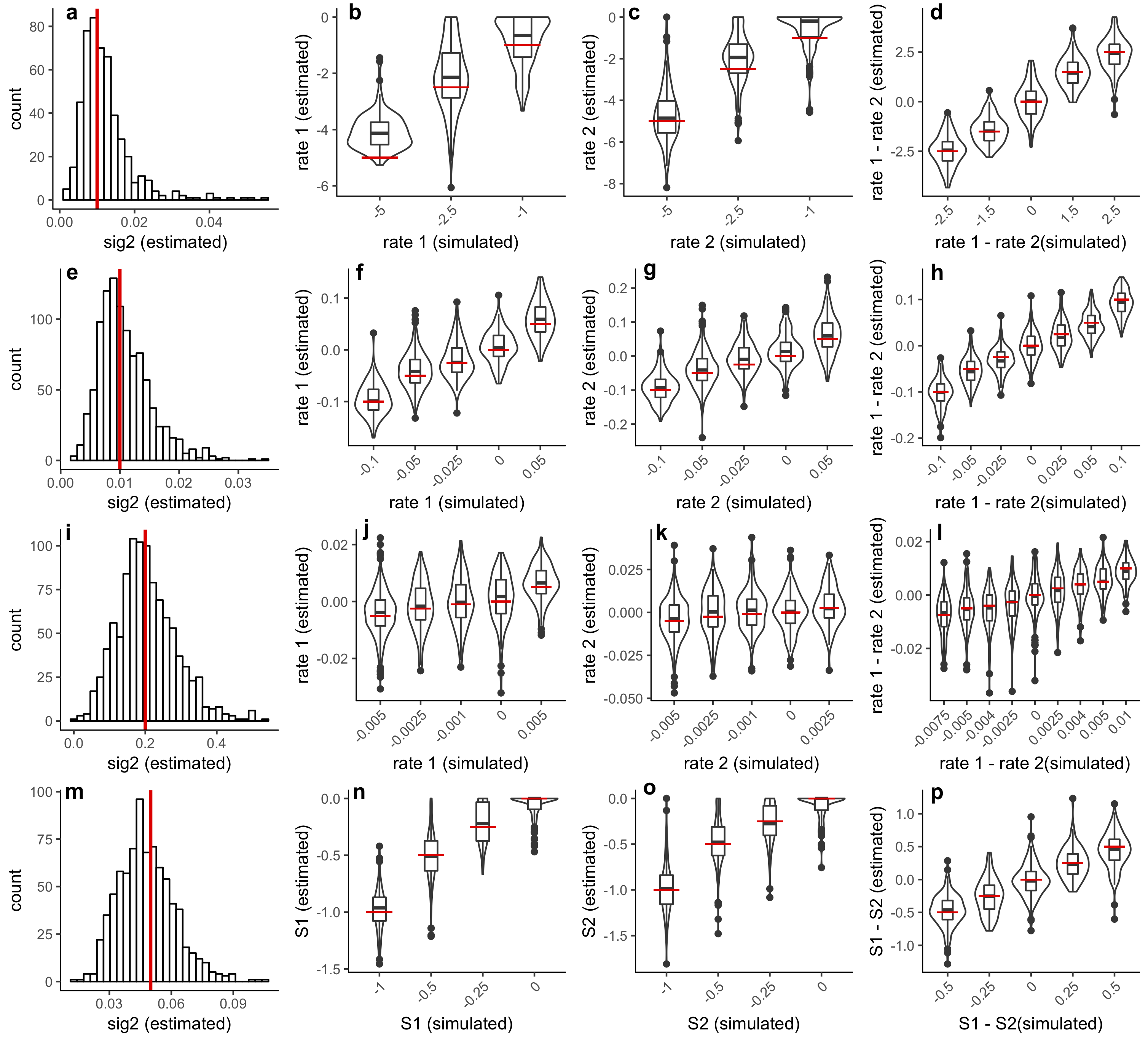

Supplement: S2 Fig — (a–d) Exponential time-dependent model. (e–h) DDexp model. (i–l) DDlin model. (m–p) MC model. In all plots, the red lines denote the parameters used to generate the simulated data (S7 Data). DD, diversity-dependent; DDexp, exponential diversity-dependent; DDlin, linear diversity-dependent; MC, matching competition; ML, maximum likelihood. (TIFF) [file pbio.3001270.s021.tiff]

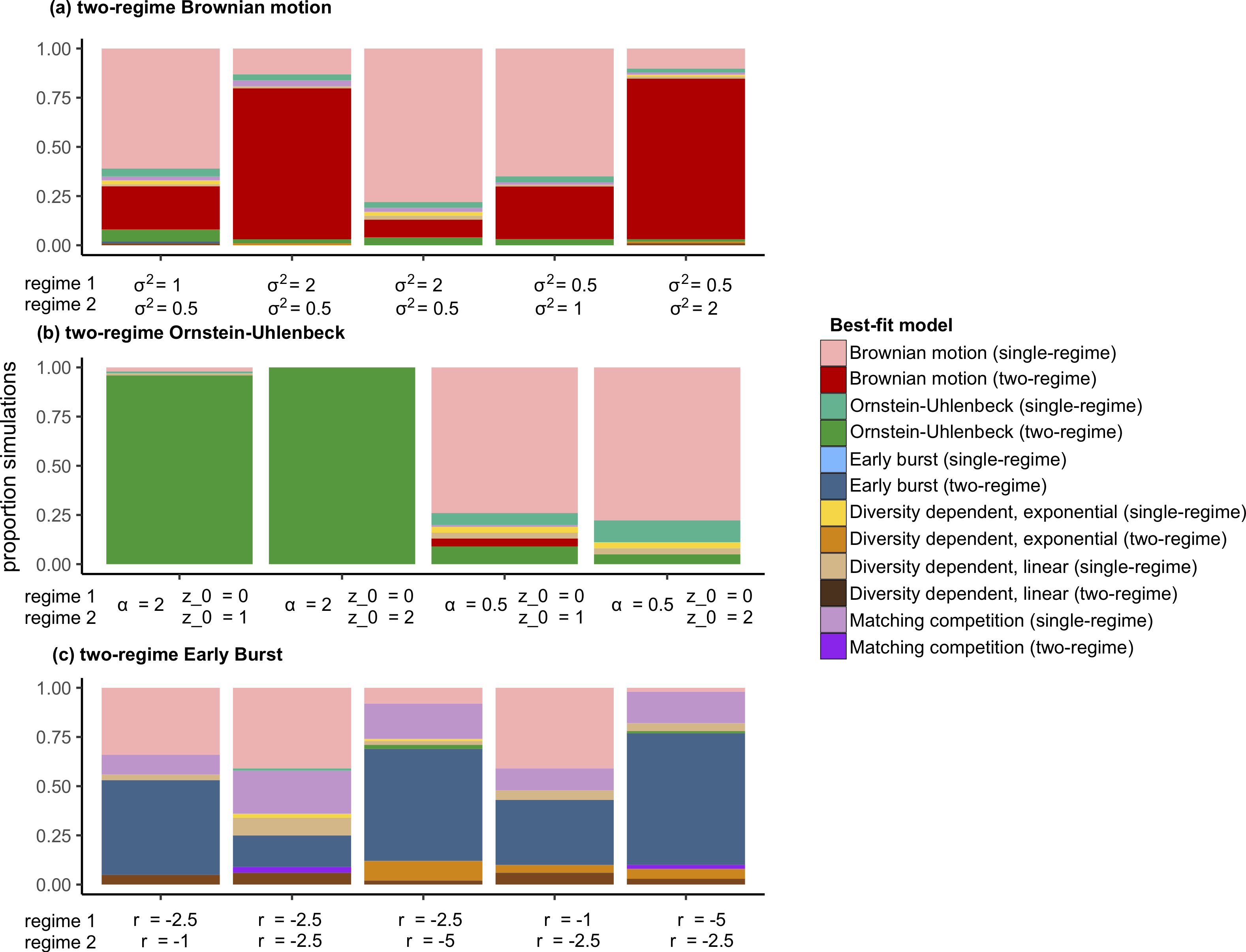

Supplement: S3 Fig — Results of model selection depicting best-fitting models for data simulated under (a) 2-regime BM, (b) 2-regime OU, and (c) 2-regime EB models across a range of parameter values (S8 Data). BM, Brownian motion; EB, early burst; OU, Ornstein–Uhlenbeck. (TIFF) [file pbio.3001270.s022.tiff]

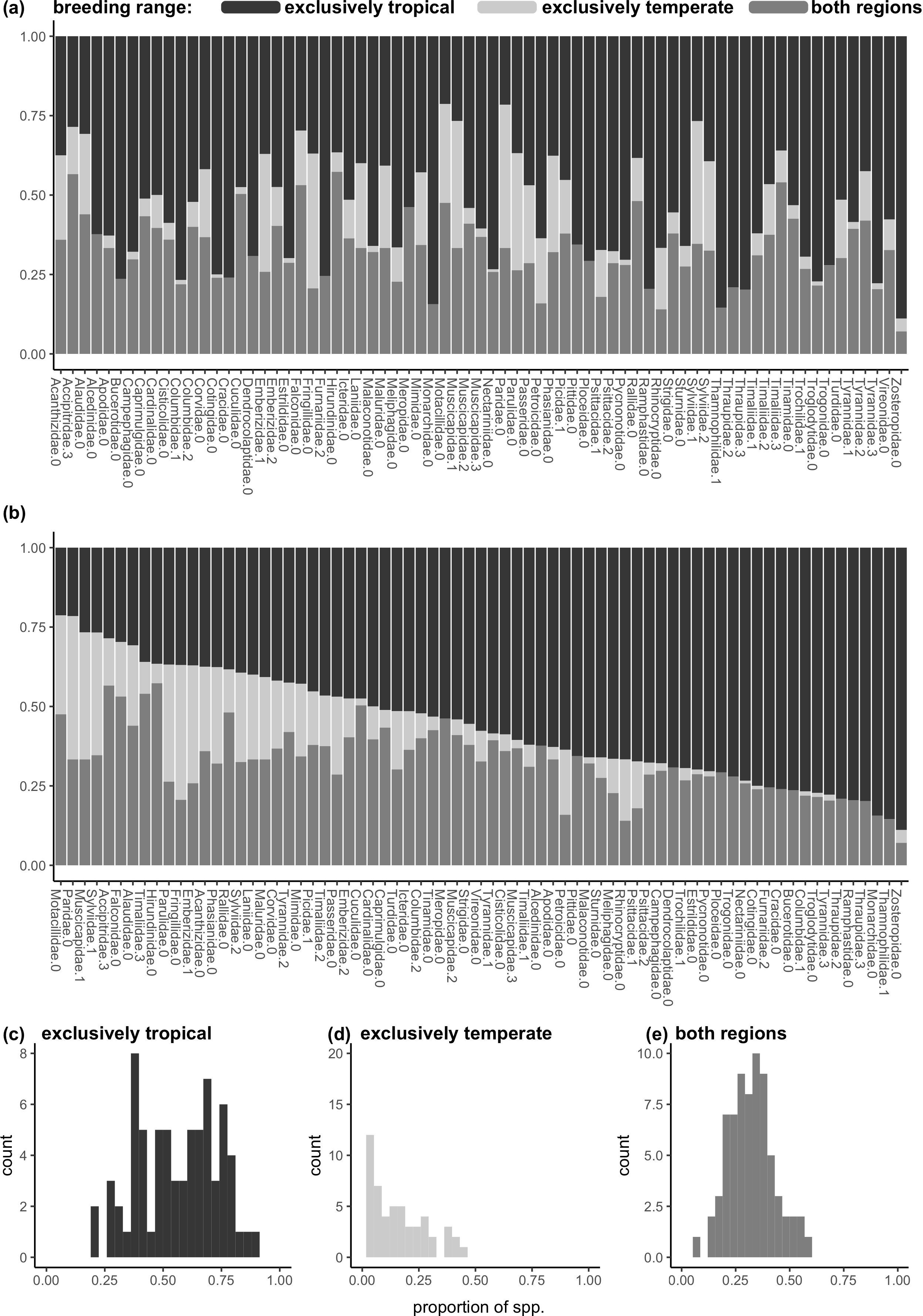

Supplement: S4 Fig — Clade-level distributions of tropical, temperate, and widespread breeding (a) sorted by clade name, (b) sorted by proportion of exclusively tropical breeding species, and (c and d) presented as separate histograms. The number following the family name indicates the subclade within that family (see Methods and S4 and S5 Data). (TIFF) [file pbio.3001270.s023.tiff]

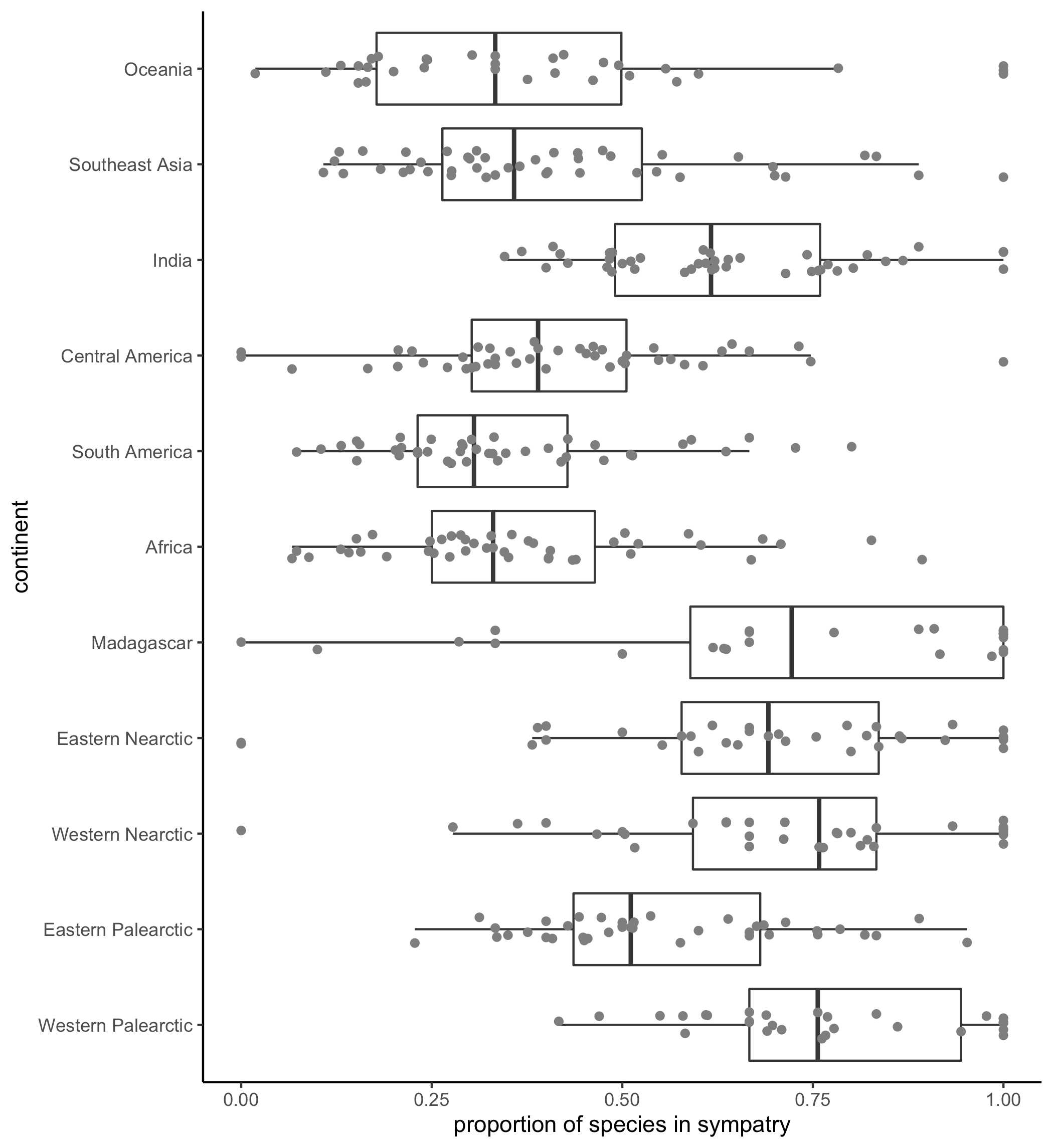

Supplement: S5 Fig — (TIFF) [file pbio.3001270.s024.tiff]

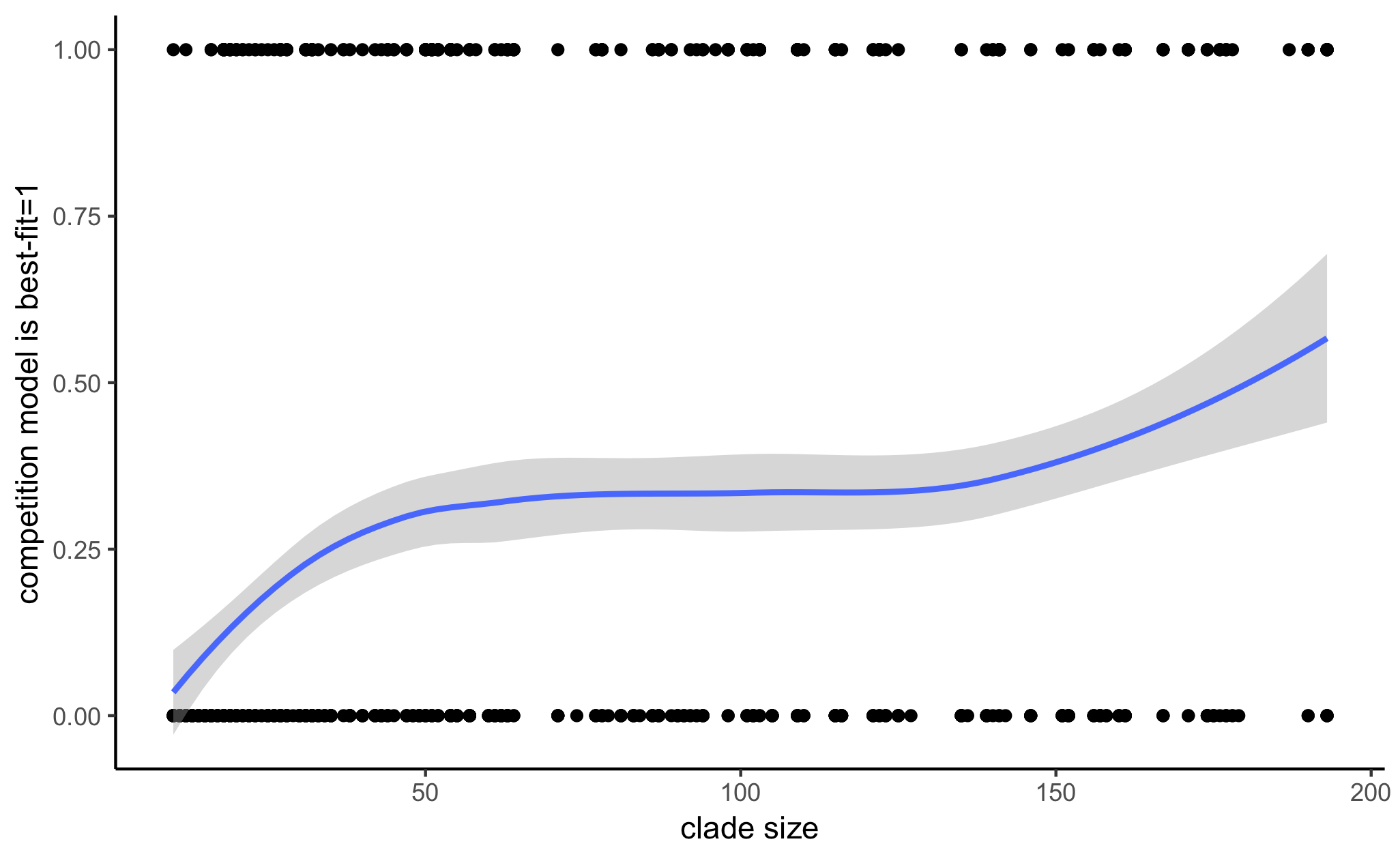

Supplement: S6 Fig — (TIFF) [file pbio.3001270.s025.tiff]

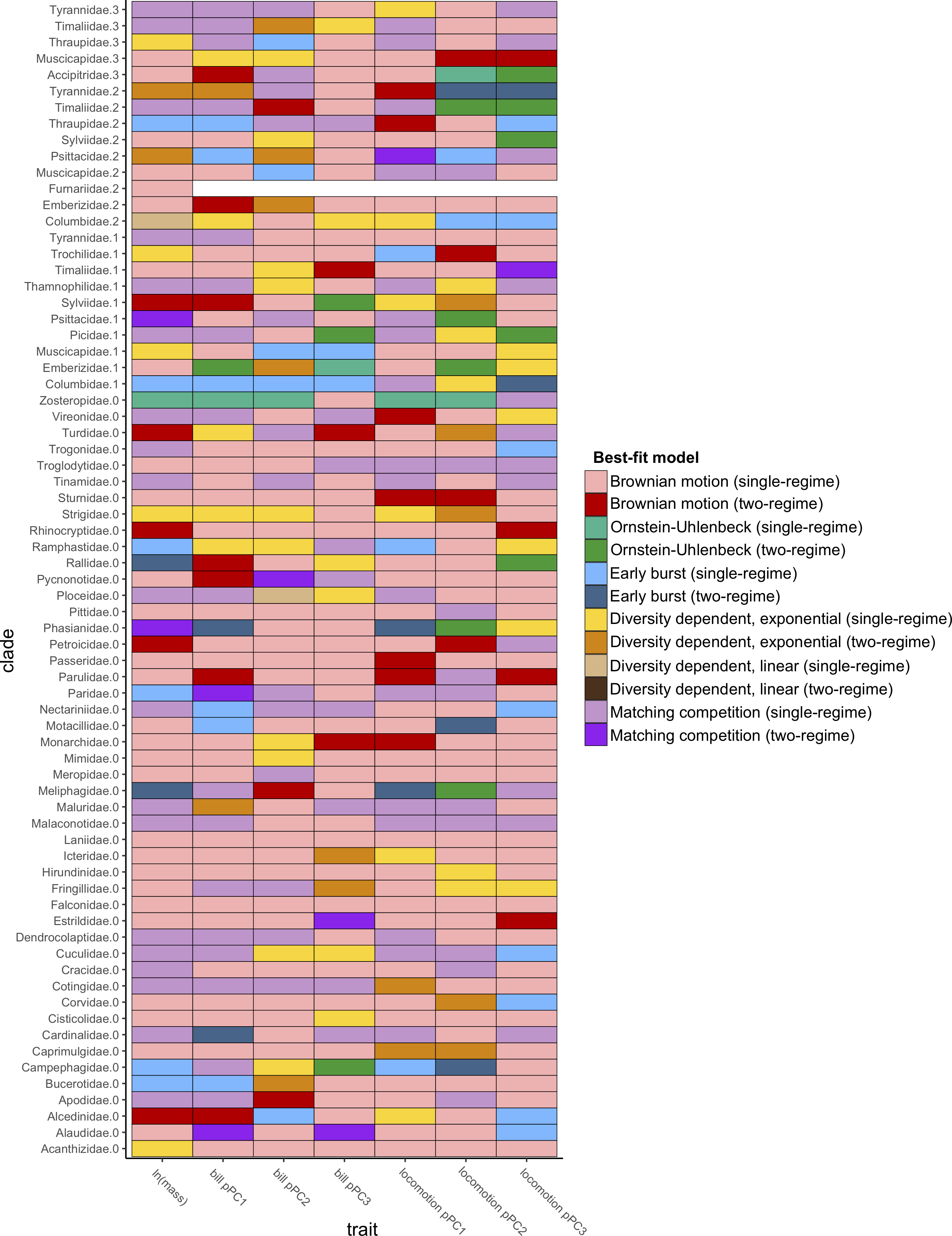

Supplement: S7 Fig — Shown is the modal best-fit model (i.e., the most common best-fit model across fits conducted on a bank of stochastic maps of ancestral biogeography across fits conducted on a bank of stochastic maps of ancestral biogeography and stochastic maps of breeding range). The number following the family name indicates the subclade within that family (see Methods and S4 and S5 Datas). (TIFF) [file pbio.3001270.s026.tiff]

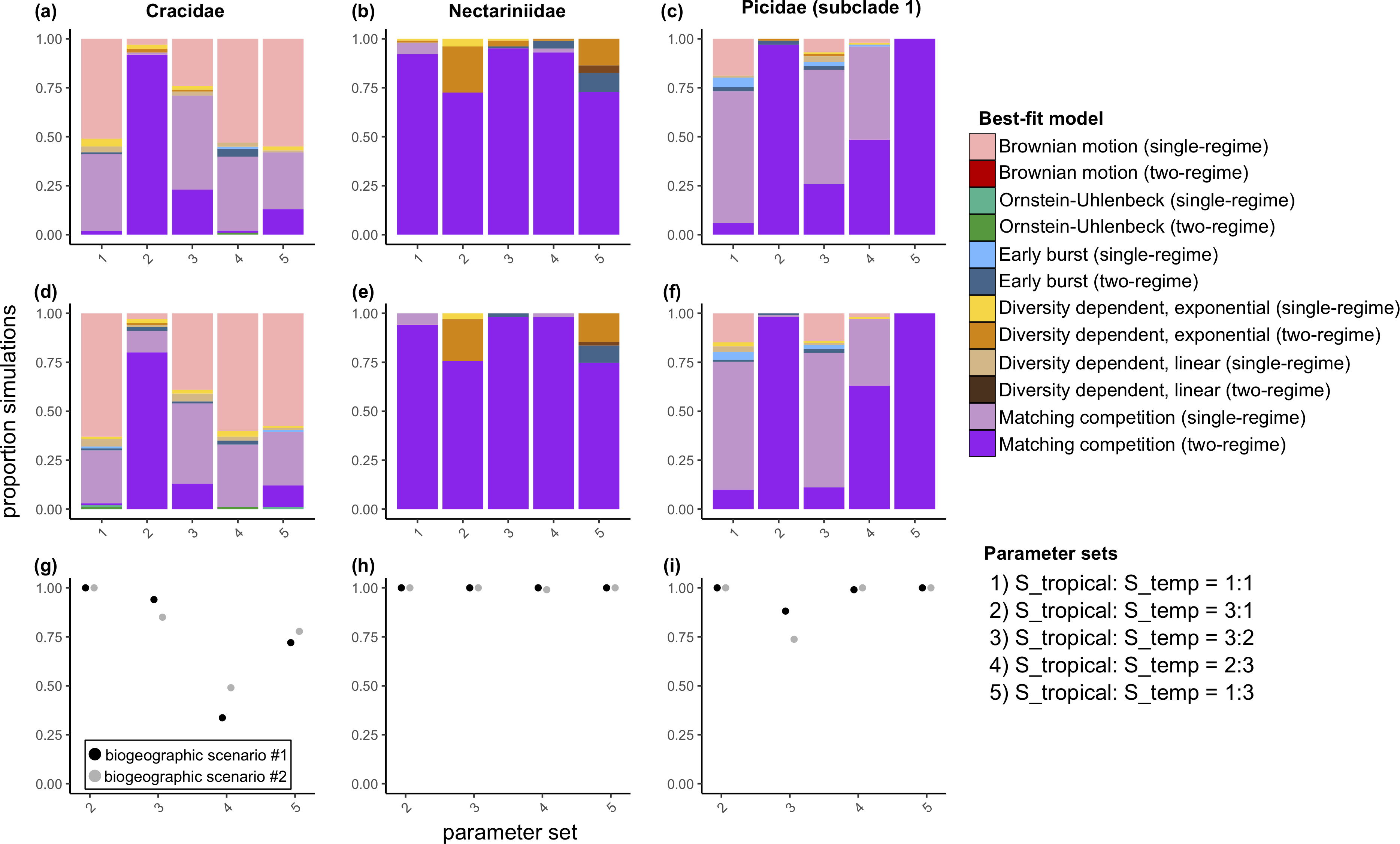

Supplement: S8 Fig — (a–c) Best-fit models for data generated under downsampled biogeographic scenario #1 (i.e., 50% of both tropical and temperate lineages set to allopatric at a continental scale). (d–f) Best-fit models for data generated under downsampled biogeographic scenario #2 (i.e., 50% of temperate lineages and 66.6% of tropical lineages set to allopatric at a continental scale). (g–i) The proportion of simulations for which MLEs of the ratio of competition from the 2-regime MC model (i.e., ln(|Stropical|/|Stemperate|)) correctly identify the direction of the difference in the strength of competition (S9 Data). MLE, maximum likelihood estimate. (TIFF) [file pbio.3001270.s027.tiff]

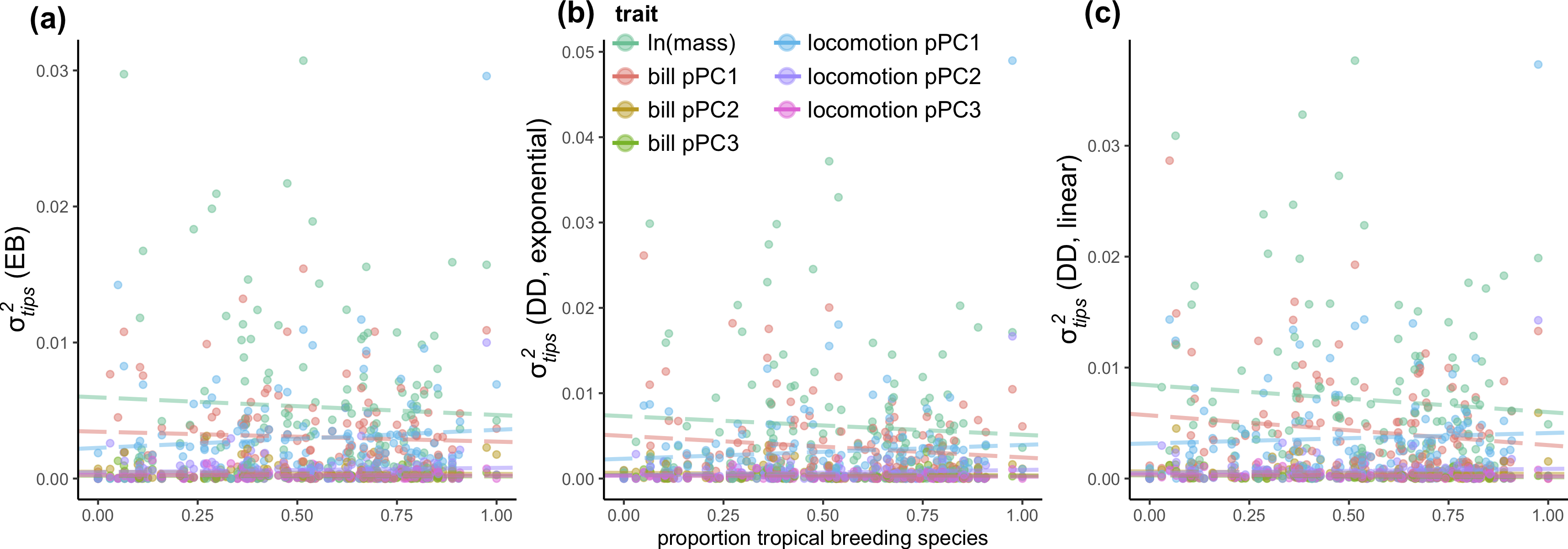

Supplement: S9 Fig — Evolutionary rates in other single-regime models (a: EB, b: DDexp, and c: DDlin) do not vary as a function of the proportion of lineages that breed in the tropics. For DD models, parameter estimates are the mean estimates across fits conducted on a bank of stochastic maps of ancestral biogeography (S2 and S3 Datas). DD, diversity-dependent; DDexp, exponential diversity-dependent; DDlin, linear diversity-dependent; EB, early burst. (TIFF) [file pbio.3001270.s028.tiff]

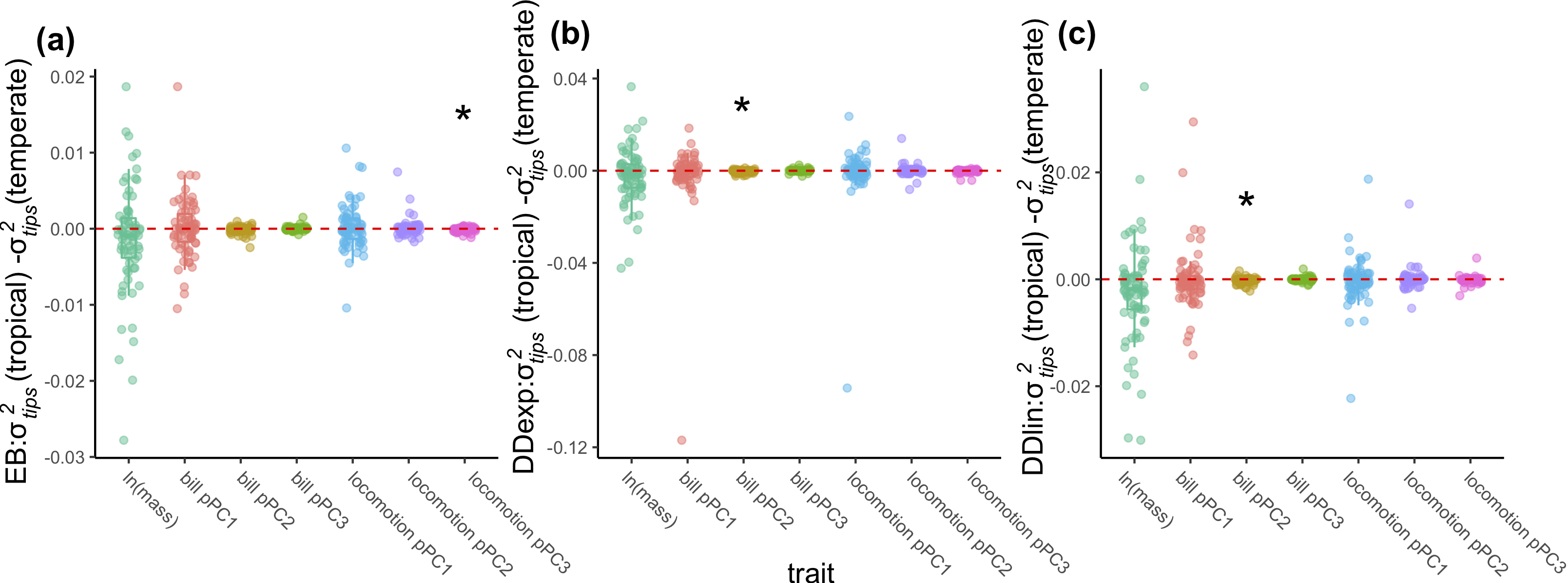

Supplement: S10 Fig — Differences between rates estimated separately on tropical and temperate taxa in 2-regime models (a: EB, b: DDexp, and c: DDlin). Shown are the mean comparisons between parameter estimates across fits conducted on a bank of stochastic maps of ancestral biogeography and stochastic maps of breeding range (i.e., tropical or temperate). Asterisks indicate statistical significance (S4 and S5 Datas). DD, diversity-dependent; DDexp, exponential diversity-dependent; DDlin, linear diversity-dependent; EB, early burst. (TIFF) [file pbio.3001270.s029.tiff]

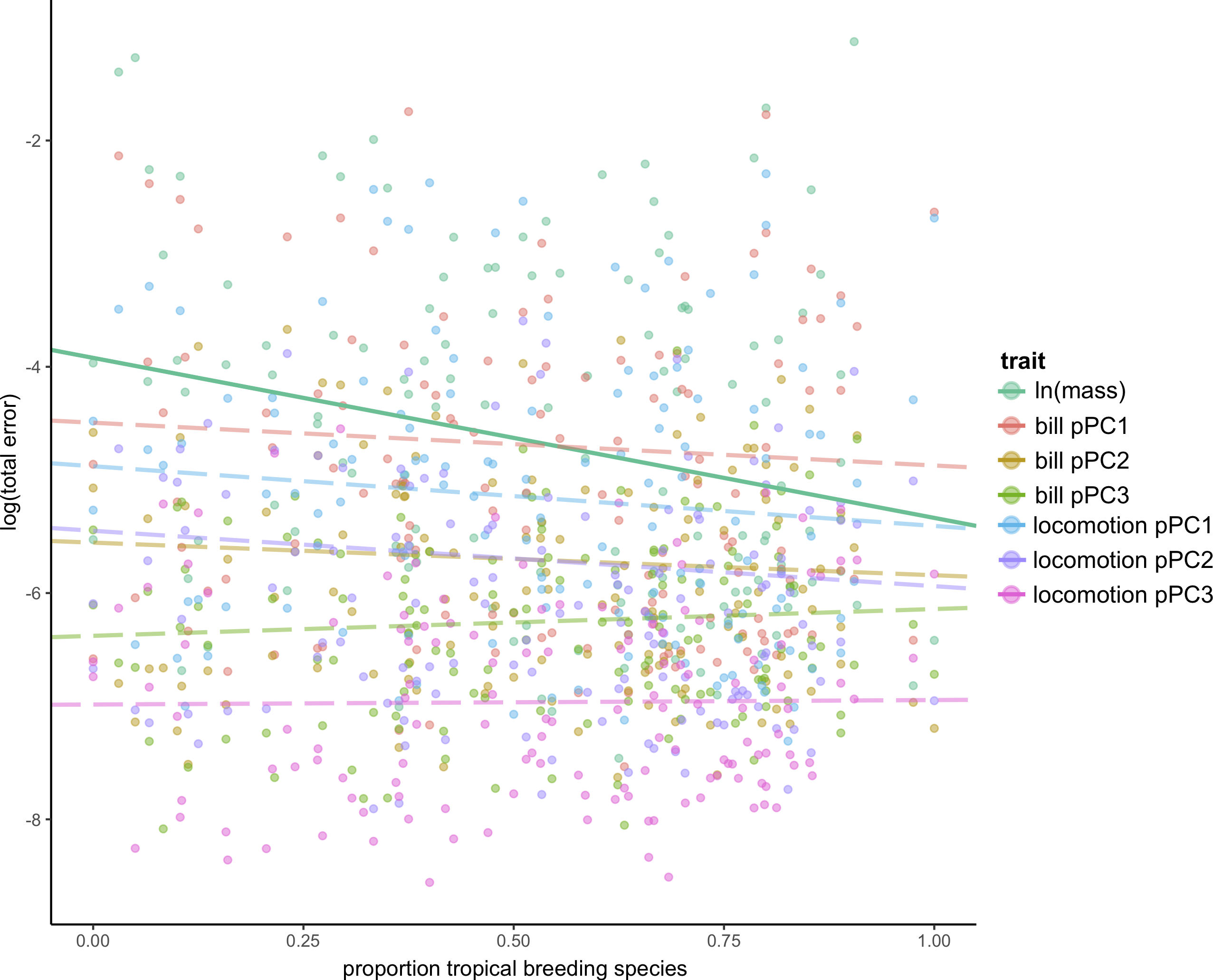

Supplement: S11 Fig — Solid lines represent statistically significant relationships (S15 Table and S10 Data). BM, Brownian motion; MLE, maximum likelihood estimate. (TIFF) [file pbio.3001270.s030.tiff]

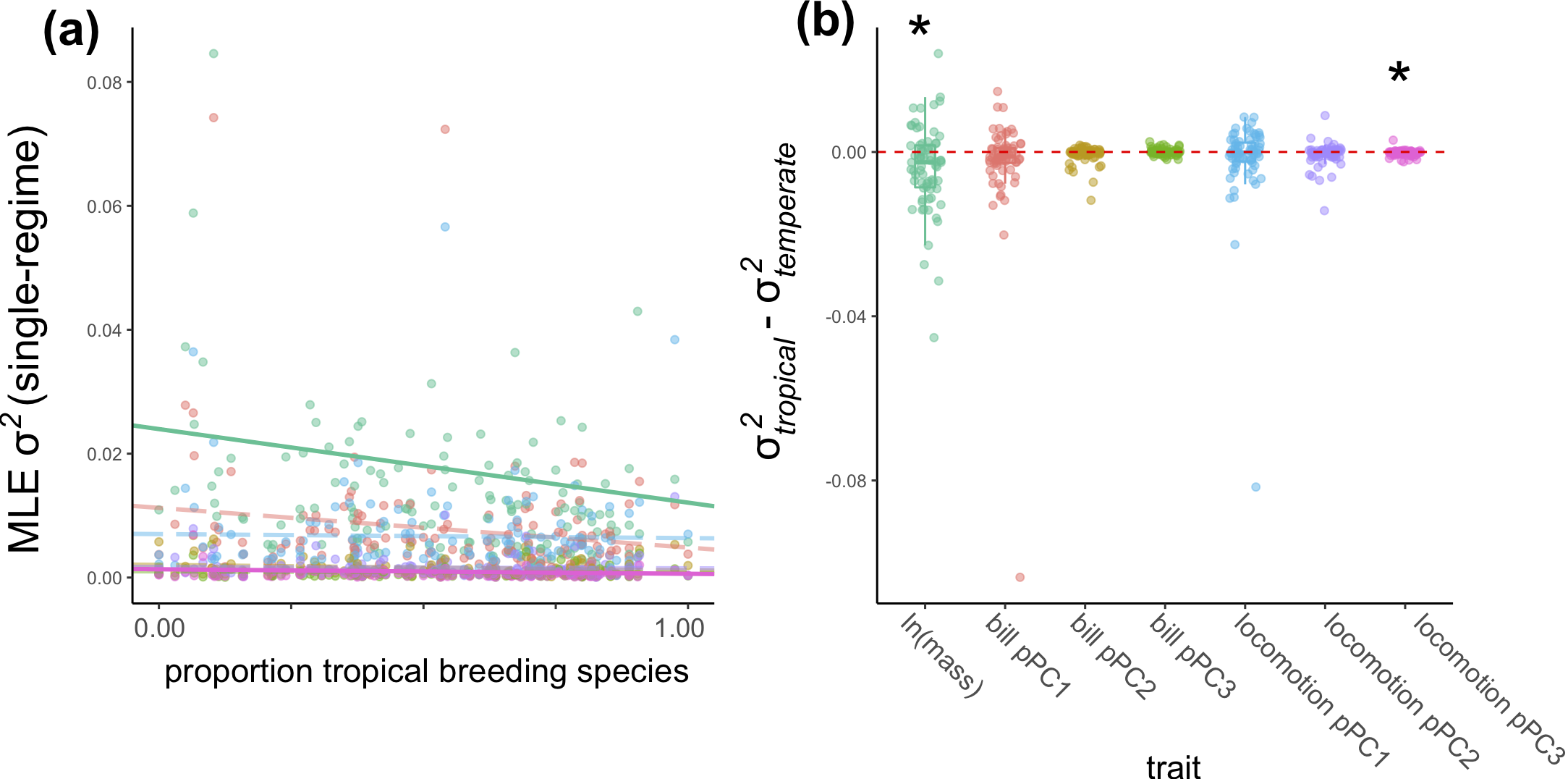

Supplement: S12 Fig — (a) There is a negative relationship between the proportion of taxa in a clade that breed in the tropics and the estimated rate of trait evolution from single-rate BM models for body mass and locomotion pPC3, but not other traits. Color of points indicate trait (as in panel b). (b). Differences between rates estimated separately on tropical and temperate taxa in 2-rate BM models are biased toward faster rates in temperate regions for body mass and locomotion pPC3, but not other traits. Shown are the mean comparisons between parameter estimates across fits conducted on a bank of stochastic maps of ancestral biogeography and stochastic maps of breeding range (i.e., tropical or temperate) (S11 Data). BM, Brownian motion; pPC, phylogenetic principal component. (TIFF) [file pbio.3001270.s031.tiff]

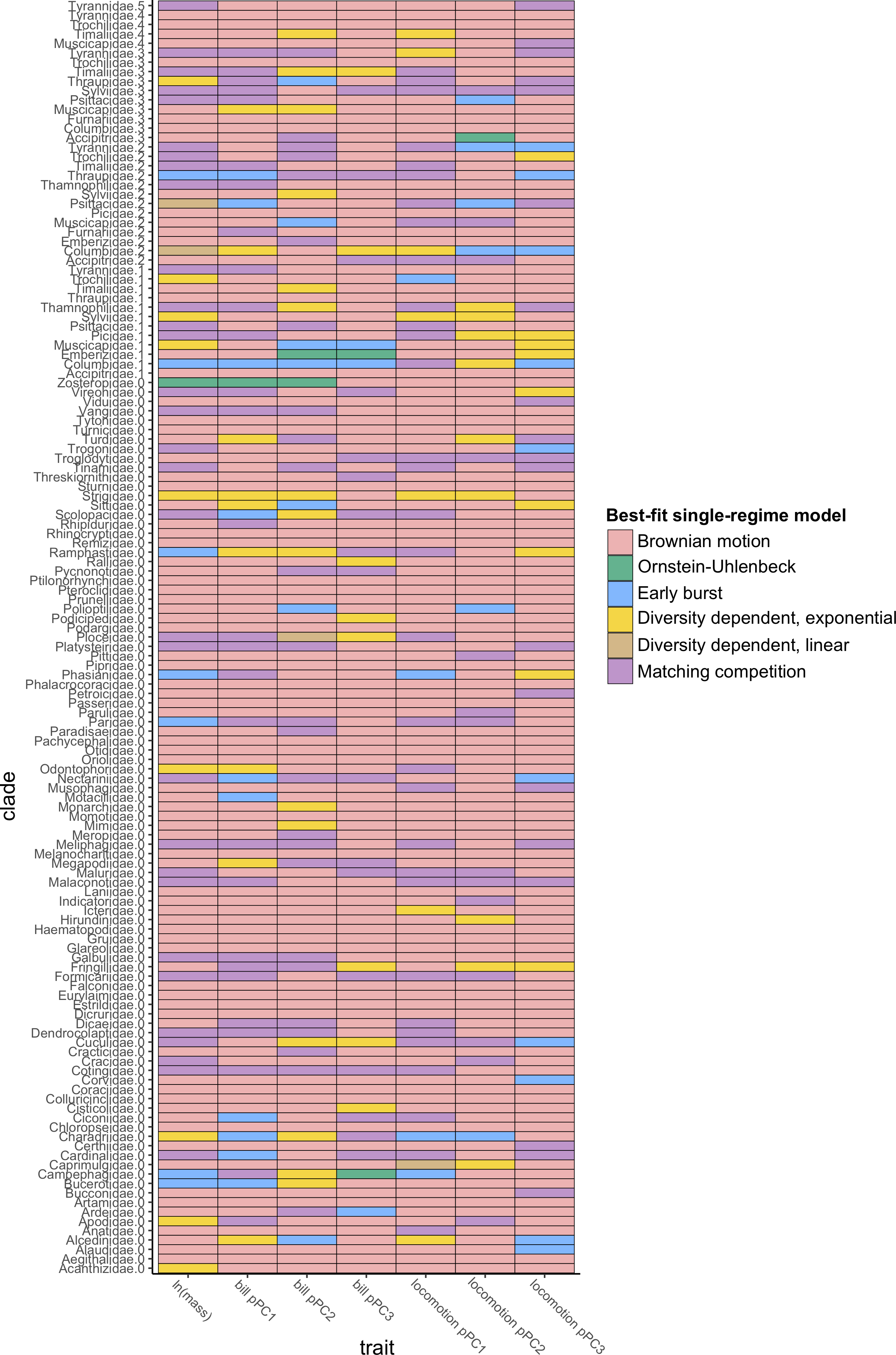

Supplement: S13 Fig — Shown is the modal best-fit model across fits conducted on a bank of stochastic maps of ancestral biogeography. The number following the family name indicates the subclade within that family (see Methods and S2 and S3 Datas). BM, Brownian motion; MC, matching competition. (TIFF) [file pbio.3001270.s032.tiff]

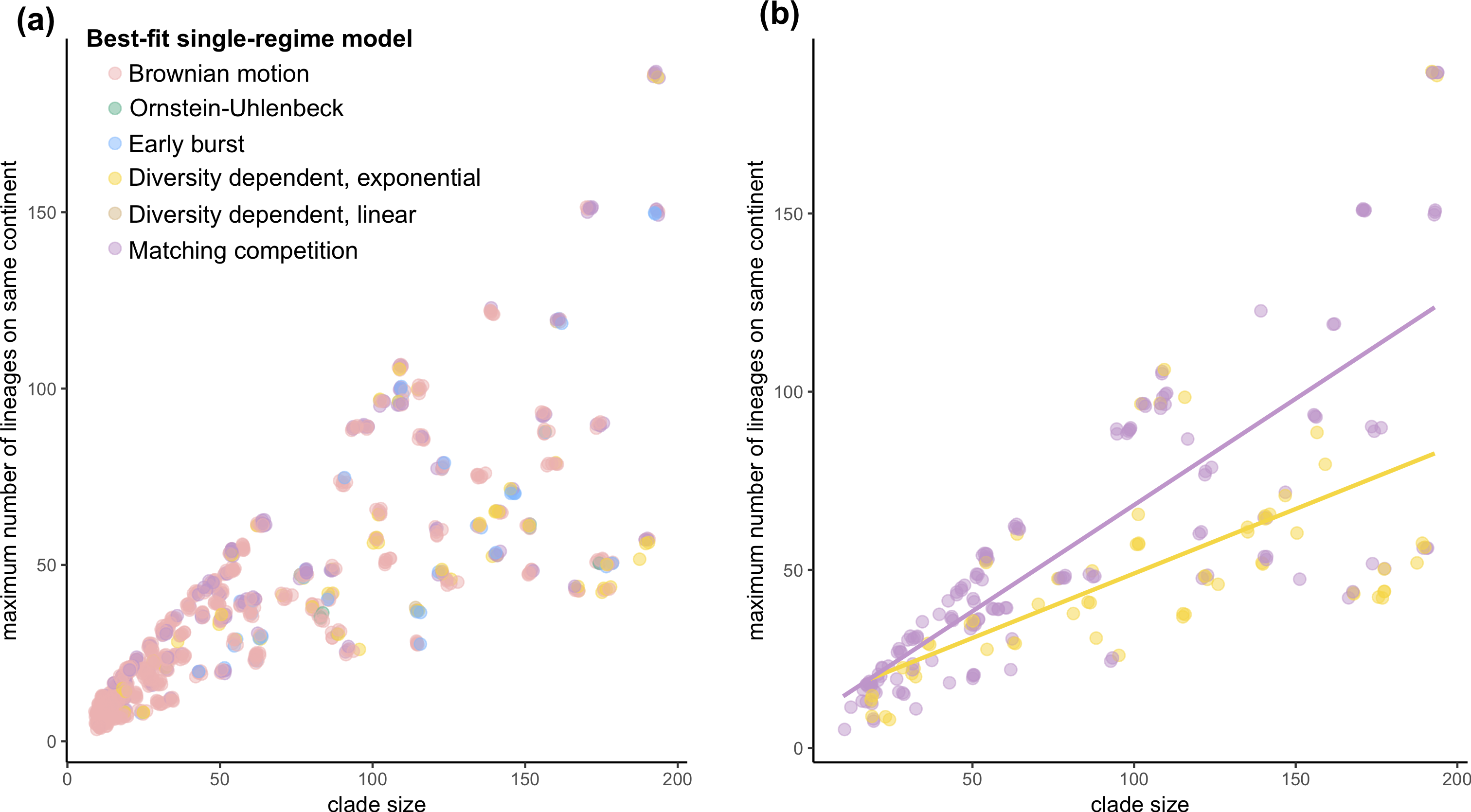

Supplement: S14 Fig — (A) All models. (B) MC and DDexp models. Each point represents a clade-by-trait combination (i.e., each clade contributes a point for each of 7 traits). In both panels, points are jittered slightly to aid visualization (S2 and S3 Datas). DDexp, exponential diversity-dependent; MC, matching competition. (TIFF) [file pbio.3001270.s033.tiff]
